# A highly diverse fungal community associated with leaves of the mangrove plant *Acanthus ilicifolius* var. *xiamenensis* revealed by isolation and metabarcoding analyses

**DOI:** 10.7717/peerj.7293

**Published:** 2019-07-09

**Authors:** Wei-Chiung Chi, Weiling Chen, Chih-Chiao He, Sheng-Yu Guo, Hyo-Jung Cha, Ling Ming Tsang, Tsz Wai Ho, Ka-Lai Pang

**Affiliations:** 1 Institute of Marine Biology and Center of Excellence for the Oceans, National Taiwan Ocean University, Keelung, Taiwan; 2 Institute of Food Science, National Quemoy University, Kinmen, Taiwan; 3 School of Biological Science, Chinese University of Hong Kong, Shatin, New Territories, Hong Kong SAR; 4 School of Biological Sciences, University of Western Australia, Perth, Australia

**Keywords:** High throughput sequencing, Terrestrial fungi, rDNA, Illumina sequencing, Culturomics, Endophytes

## Abstract

A high diversity of culturable foliar endophytic fungi is known from various mangrove plants, and the core taxa include species from *Colletotrichum*, *Pestalotiopsis*, *Phoma*, *Phomopsis*, *Sporomiella*, among others. Since a small fraction of fungi is able to grow in culture, this study investigated the diversity of fungi associated with leaves of *Acanthus ilicifolius* var. *xiamenensis* using both isolation and metabarcoding approaches. A total of 203 isolates were cultured from surface-sterilized leaves, representing 47 different fungal species: 30 species from the winter samples (104 isolates), and 26 species from the summer samples (99 isolates). Ascomycota was dominant in both types of leaf samples, while Basidiomycota was isolated only from the summer samples. *Drechslera dematioidea* (10.58%, percentage of occurrence), *Colletotrichum* sp. 3 (7.69%) and *Alternaria* sp. (7.69%) were dominant in the winter samples; *Fusarium oxysporum* (13.13%), *Diaporthe endophytica* (10.10%) and *Colletotrichum* sp. 1 (9.09%) in the summer samples. Overall, *Corynespora cassiicola* (6.90%), *F. oxysporum* (6.40%) and *Guignardia* sp. (6.40%) had the highest overall percentage of occurrence. In the metabarcoding analysis, a total of 111 operational taxonomic units (OTUs) were identified from 17 leaf samples: 96 OTUs from the winter and 70 OTUs from the summer samples. Sequences belonging to Ascomycota and Basidiomycota were detected in both samples but the former phylum was dominant over the latter. Based on read abundance, taxa having the highest percentage of occurrence included *Alternaria* sp. (3.46%), *Cladosporium delicatulum* (2.56%) and *Pyrenochaetopsis leptospora* (1.41%) in the winter leaves, and *Aureobasidium* sp. (10.72%), *Cladosporium* sp. (7.90%), *C. delicatulum* (3.45%) and *Hortaea werneckii* (3.21%) in the summer leaves. These latter four species also had the highest overall percentage of occurrence. Combining the results from both methods, a high diversity of fungi (at least 110 species) was found associated with leaves of *A. ilicifolius* var. *xiamenensis*. Many of the fungi identified were plant pathogens and may eventually cause diseases in the host.

## Introduction

Mangroves are tropical intertidal forest communities, situated at coastal areas from low to high salinities (Tomlinson, 1986). These communities host both terrestrial and marine fungi: terrestrial fungi, such as endophytic fungi, occur on the aerial parts of the plants, while marine fungi usually grow on the submerged/intertidal dead branches of the trees. Endophytic fungi inhabit plant organs for some time in their life cycle, and they can colonize internal plant tissues without causing apparent harm to the host (Petrini, 1991). Arnold (2007) revised the definition of endophytic fungi as ‘a polyphyletic group of highly diverse, primarily ascomycetous fungi, defined functionally by their occurrence within asymptomatic tissues of plants’.

For the last decade, various mangrove plants were examined for their endophytic fungal assemblages. The Ascomycota was dominant with many asexual species while the Basidiomycota was rare (De Souza Sebastianes et al., 2013; Zhou et al., 2018). Pang et al. (2008) summarized the dominant endophytic fungi of various mangrove plant species and there were several common taxa: *Sporormiella minima*, *Guignardia*/*Phyllosticta* spp., *Phoma* spp., *Diaporthe*/*Phomopsis* spp., *Cladosporium* spp., *Acremonium* spp. and *Collectotrichum* spp. *Xylaria* spp. and *Pestalotiopsis* spp. were also common (Suryanarayanan & Kumaresan, 2000; Chaeprasert et al., 2010; Xing & Guo, 2011; De Souza Sebastianes et al., 2013). Abundance and richness of endophytic fungi of mangrove plants are dependent on mangrove plant species and also their tissue types, i.e., stem, leaf or root (Xing et al., 2011; Zhou et al., 2018). *Avicennia germinans* was found to support the lowest diversity of endophytic fungi compared with *Laguncularia racemosa* and *Rhizophora mangle*, and it was concluded to be the effect of salt excreted from leaves of *A. germinans*, which inhibits spore germination (Gilbert, Mejía-Chang & Rojas, 2002). De Souza Sebastianes et al. (2013) studied endophytic fungi in branches and leaves of *Rhizophora mangle*, *Avicennia schaueriana* and *Laguncularia racemosa* and found that branches had a higher frequency of colonization and diversity than leaves. A higher number of isolates and species richness were also obtained from stems than roots in four species of Rhizophoraceae mangrove plants (Xing & Guo, 2011). Roots of mangrove plants are inhabited with terrestrial, freshwater and marine fungi (Ananda & Sridhar, 2002). Using high throughput sequencing techniques, Arfi et al. (2012) found that different fungal classes/orders were dominant in *Avicennia marina* and *Rhizophora stylosa* and between aerial and intertidal parts of the trees. Some endophytic fungi are host-specific and their diversity is seasonally varied (Suryanarayanan, Kumaresan & Johnson, 1998; Costa, Maia & Cavalcanti, 2012). Diversity of endophytic fungi increased with leaf age and some fungi may switch from an endophytic lifestyle to a saprobic one after leaf fall (Kumaresan & Suryanarayanan, 2002).

*Acanthus ilicifolius* var. *xiamenensis* is a mangrove plant distributed along the coast of southern China. The only distribution of *A. ilicifolius* var. *xiamenensis* in Taiwan is at Liuyu Township, Kinmen County with two small patches. However, their survival is under threat due to construction work for urban development. Previous studies on endophytic fungal assemblages associated with *A. ilicifolius* found that *Colletotrichum* spp. and *Phomopsis* spp. were the dominant species (Suryanarayanan & Kumaresan, 2000; Chaeprasert et al., 2010). This study investigates the cultural diversity of endophytic fungi of surface-sterilized healthy leaves of *A. ilicifolius* var. *xiamenensis* and the diversity of fungi of the same leaves using Illumina MiSeq sequencing.

## Materials and Methods

### Collection of samples

**Figure 1:**
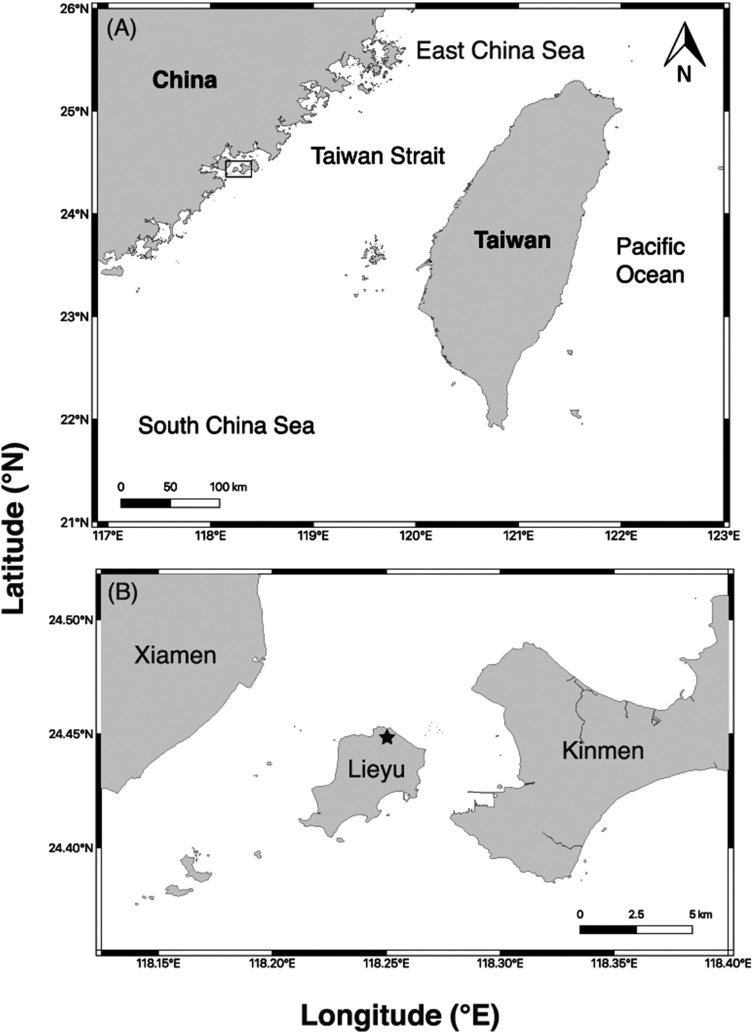
Sampling site. (A) Location of Kinmen County (box), Taiwan; (B) distribution of *Acanthus ilicifolius* var. *xiamenensis* at Lieyu Township, where the samples were collected (star).

**Figure 2:**
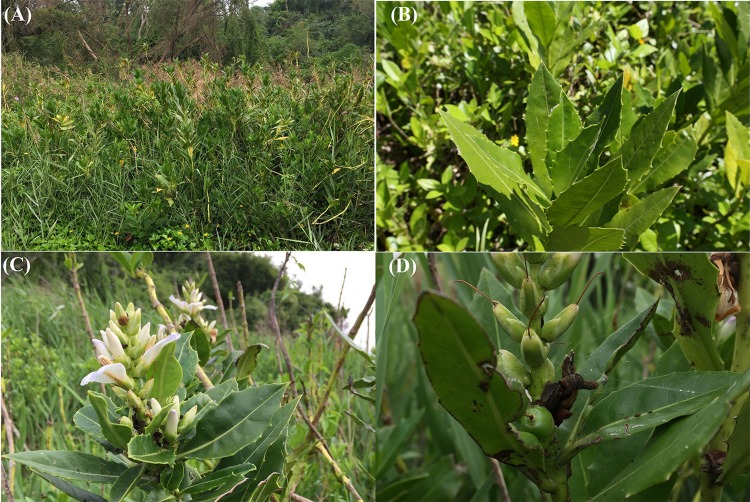
Morphology of*Acanthus ilicifolius* var. *xiamenensis*. (A) Trees, (B) healthy leaves, (C) flowers, and (D) fruits surrounded by unhealthy leaves.

The mangrove plants *Aegiceras corniculatum*, *Acanthus ilicifolius* var. *xiamenensis* and *Kandelia obovata* are present at Lieyu Township, Kinmen County, Taiwan. *A. ilicifolius* var. *xiamenensis* is the only mangrove plant growing at the sampling site at Lieyu Township and it represents the only distribution in Taiwan (Fig. 1). The characteristics of *A. ilicifolius* var. *xiamenensis* are shown in Fig. 2. Healthy leaves (i.e., for isolation of endophytic over saprobic/pathogenic fungi) were collected on 16 January (60 leaves) and 11 July (35 leaves) 2014, placed in a cool box and transported to the laboratory at National Taiwan Ocean University for immediate fungal isolation.

### Fungal isolation

Leaves were washed with tap water to remove surface dirt. Four discs (6 mm in diameter) were cut out from each leaf, surface-sterilized by immersing in 70% ethanol for 10 s and 4% sodium hypochlorite solution for 30 s, washed twice in sterile distilled water, and plated on 2% malt extract freshwater agar (MEAF, BD Bacto™; BD Biosciences, Sparks, MD, USA), supplemented with 0.5 g/L each of streptomycin sulfate (Sigma-Aldrich, MO, USA) and Penicillin G (Sigma-Aldrich, MO, USA). The inoculated plates were incubated at 25 °C and checked daily to observe fungal growth from the leaf discs for 1 month. Hyphal tips of different mycelial morphotypes from each plate (i.e., from the same leaf) were isolated and subcultured onto fresh MEAF. All cultures were kept at National Taiwan Ocean University.

### Identification of fungal isolates

All isolated cultures were grouped into different colony morphologies, and identified by comparing their ITS sequences with those deposited in the National Center for Biotechnology Information (NCBI). Mycelia for each morphotype were ground into fine powder in liquid nitrogen using a mortar and pestle. Genomic DNA was extracted using the DNeasy Plant DNA Extraction Kit (Qiagen, Germantown, MD, USA) according to the manufacturer’s instructions. ITS was amplified using the primer pairs ITS1 (or ITS5)/ITS4 (White et al., 1990). PCR reactions were performed in a 25 µL volume containing ca. 20 ng DNA, 0.2 µM of each primer, 0.2 mM of each dNTP, 2.5 mM MgCl_2_ and 1.25 U of Taq Polymerase (Invitrogen, Sao Paulo, Brazil). The amplification cycle consisted of an initial denaturation step of 95 °C for 2 min followed by 35 cycles of (a) denaturation (95 °C for 1 min), (b) annealing (54 °C for 1 min) and (c) elongation (72 °C for 1.5 min) and a final 10 min elongation step at 72 °C. The PCR products were analysed by agarose gel electrophoresis and sent to Genomics BioSci & Tech (New Taipei City, Taiwan) for sequencing. The sequences returned were checked for ambiguity and the forward/reverse strands were assembled in MEGA7 (Kumar, Stecher & Tamura, 2016). The assembled sequences were submitted to NCBI for a nucleotide BLAST search. The ITS sequences of the fungal isolates were deposited in NCBI with the accession numbers given in Table 1.

### Metabarcoding

Seventeen leaves used for the isolation described above were freeze-dried. Total genomic DNA was extracted using QIAGEN DNeasy Plant Mini Kit (Qiagen, Hilden, Germany) according to the manufacturer’s instructions. A nested PCR approach was used to amplify a region of ITS spanning from 18S to 5.8S rDNA. The first set of primers was NSA3 (5′-AAACTCTGTCGTGCTGGGGATA-3′)/NLC2 (5′-GAGCTGCATTCCCAAACAACTC-3′) (Martin & Rygiewicz, 2005) and the second set was ITS1-F_KYO1 (5′-CTHGGTCATTTAGAGGAASTAA-3′)/ITS2 (5′-GCTGCGTTCTTCATCGATGC-3′) (White et al., 1990; Toju et al., 2012). Adapters were added to the 5′ end of the primers ITS1-F_KYO1 and ITS2. PCR amplification cycle with NSA3/NLC2 primers consisted of an initial denaturation step of 94 °C for 5 min, followed by 35 cycles of 94 °C for 30 s, 55 °C for 30 s and 72 °C for 30 s, and a final 5-min elongation step at 72 °C. For ITS1-F_KYO1/ITS2, the amplification consisted of an initial denaturation step of 95 °C for 10 min, followed by 35 cycles of 95 °C for 30 s, 55 °C for 30 s and 72 °C for 30 s, and a final 72 °C for 7 min. The PCR products were analyzed by agarose gel electrophoresis. For each leaf, five successful PCR products were pooled and purified using EasyPureTM PCR Clean up/Gel Extraction Kit (Bioman, New Taipei City, Taiwan) according to manufacturer’s instructions. The purified product was shipped to Genomics (Taipei, Taiwan) for Illumina MiSeq sequencing.

**Table 1:** Fungi isolated from surface-sterilized leaves of *Acanthus ilicifolius* var. *xiamenensis* in summer and winter sampling. Identity was based on BLAST searches in NCBI and percentage of occurrence of fungi was calculated based on number of isolates.

**Isolate number (NTOU) (accession number)**	**Sequence length (bp)**	**Phylum**	**Class**	**Order**	**Family**	**Taxa**	**Maximum score**	**Coverage(%)**	**Similarity(%)**	**Matched sequence(s)**	**Occurrence (%)**		
											**Winter**	**Summer**	**Total**
4398 (MK448262)	529	Ascomycota	Dothideomycetes	Capnodiales	Teratosphaeriaceae	*Acidiella uranophila*	832	100	95	JQ904602	0.96	0.00	0.49
4330 (MK432953), 4899 (MK432954), 4902 (MK432955), 4904 (MK432956)	485–544	Ascomycota	Dothideomycetes	Pleosporales	Pleosporaceae	*Alternaria alternata*	896–1005	100	100	LC317410, MF422130	1.92	7.07	4.43
4336 (MK448263), 4368 (same colony morphology as 4336)	543	Ascomycota	Dothideomycetes	Pleosporales	Pleosporaceae	*Alternaria* sp.	1003	100	100	KY190102	7.69	0.00	3.94
4350 (MK448264)	556	Ascomycota	Dothideomycetes	Dothideales	Dothioraceae	*Aureobasidium pullulans*	1027	100	100	LC277149, LC277150	1.92	0.00	0.99
4909 (MK432957)	549	Ascomycota	Dothideomycetes	Dothideales	Dothioraceae	*Aureobasidium* sp.	1014	100	100	KF367567	0.00	1.01	0.49
4875 (MK448265)	557	Ascomycota	Dothideomycetes	Botryosphaeriales	Botryosphaeriaceae	*Botryosphaeria dothidea*	1029	100	100	KU686880	0.00	3.03	1.48
4340 (MK448266)	527	Ascomycota	Dothideomycetes	Capnodiales	Cladosporiaceae	*Cladosporium dominicanum*	974	100	100	MF472969, MF472970	1.92	0.00	0.99
4352 (MK432958), 4883 (MK432959)	524–525	Ascomycota	Dothideomycetes	Capnodiales	Cladosporiaceae	*Cladosporium* sp.	968–970	100	100	MG701131, MG572462	0.96	4.04	2.46
4372 (MK448279), 4386 (MK448280)	474–492	Ascomycota	Sordariomycetes	Glomerellales	Glomerellaceae	*Colletotrichum boninense*	837–876	100	99	FJ981604	1.92	0.00	0.99
4358 (MK448267), 4402 (MK448268)	567	Ascomycota	Sordariomycetes	Glomerellales	Glomerellaceae	*Colletotrichum hippeastri*	1001–1011	99	99	KR183779	4.81	0.00	2.46
4370 (MK432992), 4895 (MK432993), 4908 (MK432988)	515–567	Ascomycota	Sordariomycetes	Glomerellales	Glomerellaceae	*Colletotrichum* sp. 1	952–1048	100	100	MF076596, JN715846	2.88	9.09	5.91
4326 (MK448269), 4378 (MK448281), 4390 (MK448282)	536–553	Ascomycota	Sordariomycetes	Glomerellales	Glomerellaceae	*Colletotrichum* sp. 2	972–1003	100	99	HM357614	3.85	0.00	1.97
4324 (MK432994), 4356 (MK432995), 4364 (MK432989), 4903 (MK432996)	533–549	Ascomycota	Sordariomycetes	Glomerellales	Glomerellaceae	*Colletotrichum* sp. 3	985–1013	100	99–100	KX620331, KX620330, KY820893	7.69	1.01	4.43
4346 (MK432960), 4362 (MK432961), 4872 (MK432962), 4889 (MK432963)	490–533	Ascomycota	Dothideomycetes	Pleosporales	Corynesporascaceae	*Corynespora cassiicola*	898–985	99–100	99–100	FJ852578, KF266787, HM535404	5.77	8.08	6.90
4905 (MK448270), 4907 (MK448271)	546	Ascomycota	Sordariomycetes	Xylariales	Hypoxylaceae	*Daldinia eschscholtzii*	1003–1009	100	99–100	KY792621	0.00	4.04	1.97
4380 (MK432964), 4869 (same colony morphology as 4380), 4884 (MK432965), 4920 (MK432966)	550	Ascomycota	Sordariomycetes	Diaporthales	Diaporthaceae	*Diaporthe endophytica*	1011–1016	100	99–100	NR_111847	0.96	10.10	5.42
4886 (MK448272)	551	Ascomycota	Sordariomycetes	Diaporthales	Diaporthaceae	*Diaporthe longicolla*	1016	99	100	JQ754023	0.00	2.02	0.99
4382 (MK448253)	552	Ascomycota	Sordariomycetes	Diaporthales	Diaporthaceae	*Diaporthe perseae*	974	100	98.55	KC343173	0.96	0.00	0.49
4376 (MK448273), 4915 (MK448274)	550–551	Ascomycota	Sordariomycetes	Diaporthales	Diaporthaceae	*Diaporthe phaseolorum*	1002–1011	100	99	LN828206, KT964565	5.77	1.01	3.45
4334 (MK432967), 4354 (MK432968), 4400 (same colony morphology as 4334), 4404 (same colony morphology as 4334), 4878 (MK432998)	519	Ascomycota	Dothideomycetes	Pleosporales	Didymellaceae	*Didymella* sp.	948	100	99	HM012812	5.77	2.02	3.94
4901 (MK448275)	533	Ascomycota	Dothideomycetes	Dothideales	Dothioraceae	Dothioraceae sp.	894	99	97	KU892278	0.00	1.01	0.49
4410 (MK448276)	544	Ascomycota	Dothideomycetes	Pleosporales	Pleosporaceae	*Drechslera dematioidea*	1005	100	100	KY788112	10.58	0.00	5.42
4870 (MK448277), 4873 (MK448278), 4879 (MK448283), 4885 (MK448284), 4896 (MK448285), 4898 (MK448286)	467–518	Ascomycota	Sordariomycetes	Hypocreales	Nectriaceae	*Fusarium oxysporum*	863–957	100	100	MG727665, MG722826	0.00	13.13	6.40
4866 (MK432970), 4876 (MK432971), 4877 (MK432972)	488–532	Ascomycota	Sordariomycetes	Hypocreales	Nectriaceae	*Fusarium* sp.	902–983	100	100	MG562501, MG274294	0.00	8.08	3.94
4318 (MK432973), 4332 (MK432974), 4388 (MK432975), 4871 (MK432976), 4874 (MK432977)	587–613	Ascomycota	Dothideomycetes	Botryosphaeriales	Botryosphaeriaceae	*Guignardia* sp.	1085–1133	100	100	JQ341114, MF170677, LN828209, JN791605	5.77	7.07	6.40
4320 (MK432978), 4396 (MK432979)	432–523	Ascomycota	Dothideomycetes	Capnodiales	Teratosphaeriaceae	*Hortaea werneckii*	798–966	100	100	GQ334389, KY434149	3.85	0.00	1.97
4348 (MK448249)	525	Ascomycota	Sordariomycetes	Xylariales	Apiosporaceae	*Nigrospora sphaerica*	970	100	100	MH028054, MG669225	3.85	0.00	1.97
4868 (MK432980)	720	Ascomycota	Sordariomycetes	Xylariales	Xylariaceae	*Nodulisporium* sp.	1297	100	99	KR016438	0.00	2.02	0.99
4408 (MK448250)	510	Ascomycota	Dothideomycetes	Pleosporales	Phaeosphaeriaceae	*Parastagonospora phoenicicola*	846	100	97	KY173428	1.92	0.00	0.99
4914 (MK432981)	579	Ascomycota	Sordariomycetes	Amphisphaeriales	Pestalotiopsidaceae	*Pestalotiopsis microspora*	1070	100	100	KX755255	0.00	1.01	0.49
4394 (MK448260)	550	Ascomycota	Dothideomycetes	Capnodiales	Mycosphaerellaceae	*Phaeophleospora eucalypticola*	1016	100	100	NR_145123	0.96	0.00	0.49
4906 (MK432982)	618	Basidiomycota	Agaricomycetes	Polyporales	Phanerochaetaceae	*Phanerina mellea*	1136	100	99	KX752602	0.00	1.01	0.49
4917 (MK440618)	670	Basidiomycota	Agaricomycetes	Hymenochaetales	Hymenochaetaceae	*Phellinus noxius*	1218	99	99	KF233592	0.00	2.02	0.99
4406 (MK448251)	508	Ascomycota	Dothideomycetes	Pleosporales		*Phoma* sp. 1	939	100	100	KY780194	1.92	0.00	0.99
4338 (MK432990), 4366 (MK432991)	465	Ascomycota	Dothideomycetes	Pleosporales		*Phoma* sp. 2	859	100	100	JX157864	2.88	0.00	1.48
4384 (MK448252)	551	Ascomycota	Sordariomycetes	Diaporthales	Diaporthaceae	*Phomopsis asparagi*	1007	100	99	JQ613999	0.96	0.00	0.49
4918 (MK432997)	554	Ascomycota	Sordariomycetes	Diaporthales	Diaporthaceae	*Phomopsis* sp.	883	97	96	AB245060	0.00	3.03	1.48
4893 (MK432983)	517	Ascomycota	Dothideomycetes	Capnodiales	Mycosphaerellaceae	*Pseudocercospora nymphaeacea*	955	100	100	KY304491	0.00	1.01	0.49
4374 (MK448254)	518	Ascomycota	Dothideomycetes	Capnodiales	Mycosphaerellaceae	*Pseudocercospora* sp.	957	100	100	KP896027	1.92	0.00	0.99
4892 (MK448255)	532	Ascomycota	Dothideomycetes	Capnodiales	Dissoconiaceae	*Ramichloridium punctatum*	839	100	95	MF319925	0.00	2.02	0.99
4890 (MK448256), 4891 (MK448261)	518–569	Ascomycota	Dothideomycetes	Pleosporales	Phaeosphaeriaceae	*Septoriella hubertusii*	920–970	95–100	99	KT827267	0.00	2.02	0.99
4328 (MK448257)	603	Ascomycota	Dothideomycetes	Pleosporales	Lentitheciaceae	*Setoseptoria arundinacea*	1075	97	99	LC014594	0.96	0.00	0.49
4360 (MK448258)	518	Ascomycota	Dothideomycetes	Pleosporales		*Stagonosporopsis cucurbitacearum*	957	100	100	KU059901, AB714985, AB714984	0.96	0.00	0.49
4392 (MK448259)	537	Ascomycota	Dothideomycetes	Capnodiales	Teratosphaeriaceae	*Teratosphaeria capensis*	782	100	93	JN712501	4.81	0.00	2.46
4916 (MK432984)	593	Basidiomycota	Agaricomycetes	Polyporales	Polyporaceae	*Tinctoporellus epimiltinus*	1085	100	99	KY948722	0.00	2.02	0.99
4900 (MK432985)	564	Ascomycota	Sordariomycetes	Xylariales	Xylariaceae	*Xylaria* sp.	1040	99	100	JQ388255	0.00	2.02	0.99
4322 (MK432986), 4344 (MK432987)	460–513	Ascomycota	Dothideomycetes	Capnodiales	Mycosphaerellaceae	*Zasmidium citri*	845–937	100	99	GU066616	2.88	0.00	1.48

The raw sequences were filtered with a phred score ≥Q29 (a base call accuracy of ≥99.87%). The raw reads were paired into single reads and adaptors, primers and barcode sequences were removed using the QIIME script “split_library.py” (Caporaso et al., 2010). Clustering was performed using uclust v1.2.22q (Edgar, 2010) in QIIME 1.9.0 (Caporaso et al., 2010). The reads were processed with UCHIME (Edgar et al., 2011) to reject chimeric sequences. Picking of Operation Taxonomic Units (OTUs) and taxonomic assignments were performed with an open-reference OTU picking approach against the UNITE database in QIIME 1.9.0 (Caporaso et al., 2010). A similarity threshold of 97% was adopted. Taxonomic assignment of representative OTUs was run at a 0.97 confidence threshold against the UNITE ITS1 database with UNITE 7.2 reference OTU database (“UNITE+INSD” dataset) using the assignTaxonomy method (Kõljalg et al., 2013).

### Statistical analysis

Total number of isolates (total abundance, N), Richness (total number of taxa in the community, S), Species Richness (Margalef), Shannon–Wiener Diversity Index, Pielou’s Evenness and Simpson Diversity Indices (Simpson’s Index, Simpson’s Index of Diversity, Simpson’s Reciprocal Index) were calculated in Microsoft Excel by first computing the variables of the equations and then using the math operators to calculate the different indices.

Rarefaction and extrapolation sampling curves were computed and plotted to estimate sample completeness (sample coverage) in R package iNEXT (iNterpolation/EXTrapolation) with the 95% lower and upper confidence limits for the isolation and metabarcoding data (Hsieh, Ma & Chao, 2016). A principle component analysis (PCA) was calculated by the R software using the R function prcomp() (R Core Team, 2013).

## Results

### Diversity of culturable fungi

A total of 203 isolates were cultured from leaves of *Acanthus ilicifolius* var. *xiamenensis* collected in January and July 2014 at Kinmen Township, Taiwan and ITS of the representative isolate for each morphotype was sequenced (Tables 1–2). The fungi were identified down to species level when the BLAST search results had a high percentage coverage and identity in NCBI; otherwise, they were given an identity at the genus/family level.

A total of 104 and 99 isolates were cultured from the winter (January) and summer (July) samples, representing 30 and 26 fungal species, respectively (Tables 1–2). Nine species were common between the two sampling times, therefore, 47 different fungal species were isolated from leaves of *A. ilicifolius* var. *xiamenensis*. The higher percentage of occurrence (Table 1) in the winter samples included *Drechslera dematioidea* (10.58%), *Colletotrichum* sp. 3 (7.69%) and *Alternaria* sp. (7.69%); and in the summer samples, *Fusarium oxysporum* (13.13%), *Diaporthe endophytica* (10.10%), *Colletotrichum* sp. 1 (9.09%), *Fusarium* sp. (8.08%), *Corynespora cassiicola* (8.08%), *Guignardia* sp. (7.07%) and *Alternaria alternata* (7.07%). Overall, *C. cassiicola* (6.90%), *F. oxysporum* (6.40%), *Guignardia* sp. (6.40%), *Colletotrichum* sp. 1 (5.91%), *D. endophytica* (5.42%) and *D. dematioidea* (5.42%) had the highest percentage of occurrence.

**Figure 3:**
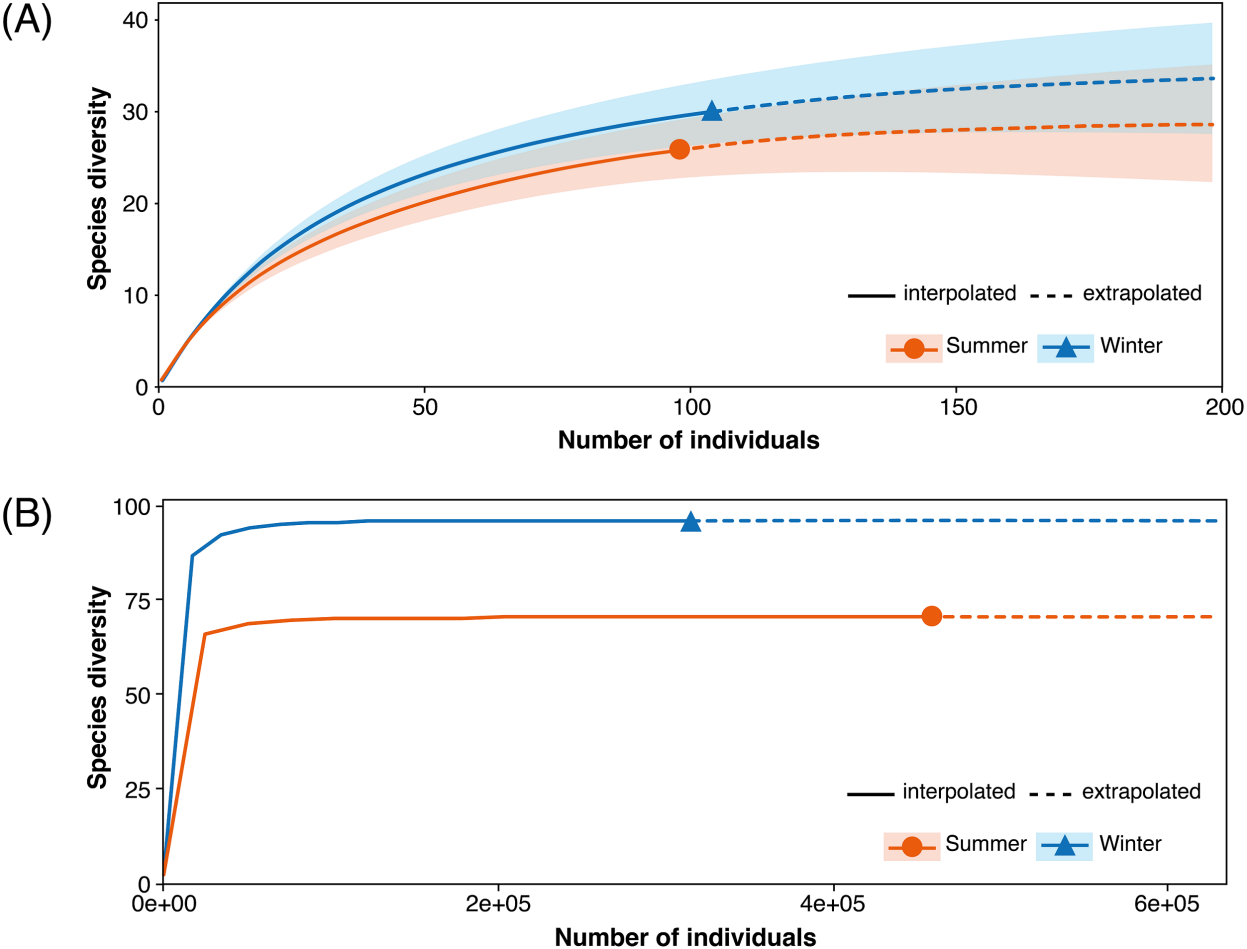
Sample-size-based rarefaction and extrapolation sampling curves. (A) Isolation and (B) metabarcoding studies of endophytic fungi associated with *Acanthus ilicifolius* var. *xiamenensis*.

Diversity indices were calculated for the fungal communities in the winter and summer samples (Table 2). The fungal community in the winter samples had a higher species richness of 6.24 (Margalef) and a higher diversity of 3.15 (Shannon–Wiener Diversity Index) than that in the summer samples (5.44 and 2.92, respectively). The Margalef and Shannon–Wiener Diversity indices with the data combining the two seasons were 8.66 and 3.83, respectively. The rarefaction and extrapolation analysis suggested that species diversity was projected to be higher in the winter samples than in the summer samples but both samples did not reach species saturation (Fig. 3A).

**Table 2:** Diversity indices of fungi associated with leaves of *Acanthus ilicifolius* var. *xiamenensis* using culture and metabarcoding analysis.

	**Culture**			**Metabarcoding analysis**		
	**Winter**	**Summer**	**Total**	**Winter**	**Summer**	**Total**
**Total No. of isolates/reads (Total Abundance), N**	104	99	203	314692	458993	773685
**Richness (Total number of Taxa in the community), S**	30	26	47	96	70	111
**Species Richness (Margalef): d = (S−1)/ln(N)**	6.24	5.44	8.66	7.50	5.29	8.11
**Shannon-Wiener Diversity Index: H′= −Σ[Pi ln(Pi)]**	3.15	2.92	3.83	1.98	2.09	2.28
**Pielou’s Evenness: J′= H′/ln(S)**	0.93	0.90	0.99	0.44	0.49	0.49
**Simpson Diversity Indices:**						
**Simpson’s Index: D = Σ(Pi2)**	0.05	0.07	0.29	0.29	0.23	0.18
**Simpson’s Index of Diversity: 1−D = 1−Σ(Pi2)**	0.95	0.93	0.71	0.72	0.78	0.82
**Simpson’s Reciprocal Index: 1/D**	19.52	14.74	3.47	3.51	4.45	5.60

**Figure 4:**
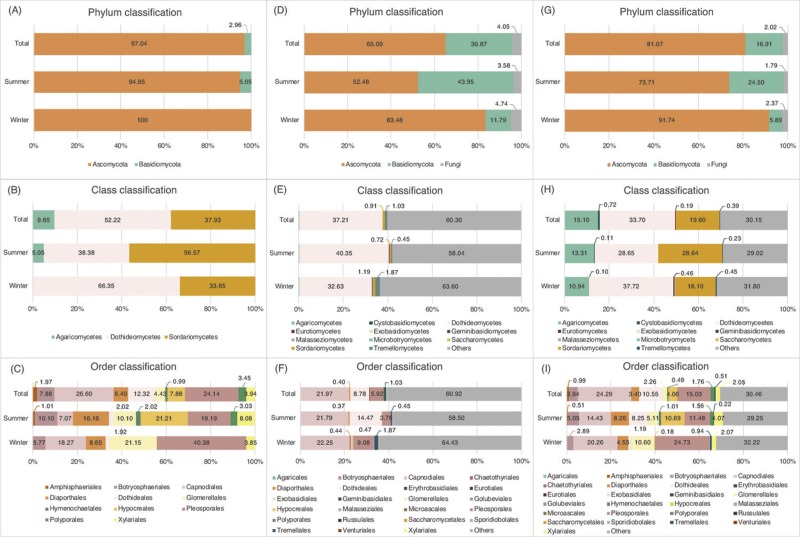
Percentage of occurrence of fungi associated with leaves of *Acanthus ilicifolius.* var. *xiamenensis*. Isolation method: (A) phylum, (B) class, and (C) order classification; metabarcoding analysis: (D) phylum, (E) class, and (F) order classification; both isolation and metabarcoding approaches: (G) phylum, (H) class, and (I) order classification.

Figures 4A–4C show the taxonomic composition of the cultured fungi at different taxonomic levels. In the winter (January 2014), only Ascomycota was isolated with no Basidiomycota; in the summer, Basidiomycota had a ∼5% occurrence (Fig. 4A). At the class level, Dothideomycetes and Sordariomycetes were the dominant classes in both seasons and Agaricomycetes was only isolated from the summer samples (Fig. 4B). At the ordinal level, the richness of fungi in summer was higher than that in winter. Seven orders Botryosphaeriales, Capnodiales, Diaporthales, Dothideales, Glomerellales, Pleosporales and Xylariales were common between the sampling times but varying in abundance (Fig. 4C). Amphisphaeriales and Hypocreales were not isolated in winter.

### Metabarcoding analysis

Seventeen samples (leaves) were analyzed by the metabarcoding analysis: 10 for the winter and 7 for the summer samples. A total of 773685 reads were obtained after QIIME analysis, including 314692 reads from the winter samples ranging from 2169 to 54606 reads, and 458993 reads from the summer samples ranging from 39151 to 93295 reads (Table 2). From the set of 17 samples, a total of 111 OTUs were identified, from which 86 could be referred to the generic level and 25 to the family level or above, including 96 OTUs (76 OTUs identified at the genus level) from the winter and 70 OTUs (55 OTUs identified at the genus level) from the summer samples (Table 3). Fifty-five OTUs (41 OTUs identified at the genus level) were common between the two seasons.

Figures 4D–4F shows the proportions of the different taxa at the phylum, class and order levels. Both Ascomycota and Basidiomycota were recovered at proportions of 52.5% (percentage of occurrence based on read number) and 44.0% from the summer and 83.5% and 11.8% from the winter samples, respectively, with a higher proportion of basidiomycetous sequences in the summer samples (Fig. 4D). Overall, Ascomycota (65.1%) was dominant over Basidiomycota (30.9%).

At the class level, 11 different fungal classes were obtained from both the winter and summer samples (Fig. 4E). Seven classes were common between the samples: Agaricomycetes, Cystobasidiomycetes, Dothideomycetes, Eurotiomycetes, Microbotryomycetes, Sordariomycetes and Tremellomycetes. Dothideomycetes was the dominant class in both winter and summer samples (32.6% and 40.4%, respectively). Other classes only constituted less than 2% of the sequences, excluding those only referred to the phylum level (‘Others’). Exobasidiomycetes and Geminibasidiomycetes were only recovered from the winter samples and likewise, Malasseziomycetes and Saccharomycetes in the summer samples. The proportion of the different major classes overall was similar to that of the individual winter and summer samples.

Twenty-two and nineteen different fungal orders were identified in the winter and summer samples, respectively (Fig. 4F). Agaricales, Botryosphaeriales, Capnodiales, Chaetothyriales, Diaporthales, Dothideales, Erythrobasidiales, Eurotiales, Glomerellales, Hypocreales, Pleosporales, Polyporales, Russulales, Sporidiobolales, Tremellales and Xylariales were recovered from both samples but varying in abundance, excluding the sequences only identified above the order level (‘Others’). The dominant orders in the winter samples were Capnodiales (22.2%), Pleosporales (9.1%) and Tremellales (1.9%). Capnodiales (21.8%) was also the most dominant order in the summer samples, followed by Dothideales (14.5%) and Pleosporales (3.8%). Exobasidiales, Geminibasidiales, Golubeviales, Microascales and Venturiales were only found in the winter samples and Malasseziales and Saccharomycetales in the summer samples; these orders exclusive to their respective sample type only constituted a low sequence abundance (<0.1%). Combining the data from the two seasons, the dominant orders were Capnodiales (22.0%), Dothideales (8.8%) and Pleosporales (5.9%).

**Table 3:** Fungal diversity associated with leaves of *Acanthus ilicifolius* var. *xiamenensis* in summer and winter samples recovered from metabarcoding analysis. Percentage of occurrence of fungi was calculated based on number of reads.

**Phylum**	**Class**	**Order**	**Family**	**Taxon**	**% Occurrence**		
					**Winter**	**Summer**	**Total**
Ascomycota	Sordariomycetes	Hypocreales	*Incertae sedis*	*Acremonium polychromum*	0.040	0.031	0.035
Basidiomycota	Agaricomycetes	Agaricales		Agaricales	0.020	0.023	0.022
Basidiomycota	Agaricomycetes			Agaricomycetes	0.166	0.136	0.148
Ascomycota	Dothideomycetes	Pleosporales	Pleosporaceae	*Alternaria* sp.	3.463	0.122	1.481
Ascomycota	Dothideomycetes	Botryosphaeriales	Aplosporellaceae	*Aplosporella yalgorensis*	0.009	0.000	0.004
Ascomycota	Sordariomycetes	Xylariales	Apiosporaceae	*Arthrinium* sp.	0.007	0.002	0.004
Ascomycota				Ascomycota	49.555	11.377	26.906
Ascomycota	Eurotiomycetes	Eurotiales	Aspergillaceae	*Aspergillus penicillioides*	0.008	0.000	0.003
Ascomycota	Eurotiomycetes	Eurotiales	Aspergillaceae	*Aspergillus* sp.	0.093	0.016	0.047
Ascomycota	Dothideomycetes	Dothideales	Aureobasidiaceae	*Aureobasidium* sp.	0.344	10.715	6.496
Ascomycota	Dothideomycetes	Dothideales	Aureobasidiaceae	*Aureobasidium thailandense*	0.000	0.064	0.038
Basidiomycota				Basidiomycota	9.308	43.089	29.349
Basidiomycota	Agaricomycetes	Polyporales	Meruliaceae	*Bjerkandera adusta*	0.023	0.032	0.029
Basidiomycota	Tremellomycetes	Tremellales	Bulleraceae	*Bullera unica*	0.044	0.000	0.018
Ascomycota	Dothideomycetes	Capnodiales		Capnodiales	15.762	5.083	9.427
Ascomycota	Dothideomycetes	Chaetothyriales	Herpotrichiellaceae	*Capronia semi-immersa*	0.036	0.035	0.035
Ascomycota	Dothideomycetes	Capnodiales	Cladosporiaceae	*Cladosporium delicatulum*	2.561	3.449	3.088
Ascomycota	Dothideomycetes	Capnodiales	Cladosporiaceae	*Cladosporium* sp.	0.189	7.901	4.764
Ascomycota	Dothideomycetes	Capnodiales	Cladosporiaceae	*Cladosporium sphaerospermum*	0.029	0.026	0.027
Ascomycota	Sordariomycetes	Glomerellales	Glomerellaceae	*Colletotrichum brasiliense*	0.011	0.063	0.042
Ascomycota	Sordariomycetes	Glomerellales	Glomerellaceae	*Colletotrichum gloeosporioides*	0.013	0.063	0.042
Ascomycota	Dothideomycetes	Pleosporales	Coniothyriaceae	*Coniothyrium sidae*	0.099	0.011	0.047
Basidiomycota	Tremellomycetes	Tremellales	Tremellaceae	*Cryptococcus dimennae*	0.073	0.000	0.030
Ascomycota	Dothideomycetes	Chaetothyriales	Cyphellophoraceae	*Cyphellophora sessilis*	0.086	0.000	0.035
Basidiomycota	Tremellomycetes	Tremellales	Bulleribasidiaceae	*Derxomyces* sp.	0.003	0.000	0.001
Ascomycota	Dothideomycetes	Capnodiales	Teratosphaeriaceae	*Devriesia* sp.	0.051	0.000	0.021
Ascomycota	Sordariomycetes	Diaporthales		Diaporthales sp. 1	0.332	0.157	0.228
Ascomycota	Sordariomycetes	Diaporthales		Diaporthales sp. 2	0.099	0.167	0.139
Ascomycota	Dothideomycetes	Pleosporales	Didymellaceae	*Didymella* sp.	0.000	0.014	0.009
Ascomycota	Dothideomycetes	Pleosporales	Didymosphaeriaceae	Didymosphaeriaceae	1.017	0.016	0.423
Basidiomycota	Tremellomycetes	Tremellales	Bulleribasidiaceae	*Dioszegia* sp.	0.367	0.002	0.150
Basidiomycota	Tremellomycetes	Tremellales	Bulleribasidiaceae	*Dioszegia takashimae*	0.113	0.032	0.065
Ascomycota	Dothideomycetes	Dothideales		Dothideales	0.000	0.491	0.291
Ascomycota	Dothideomycetes			Dothideomycetes	0.568	0.286	0.401
Basidiomycota	Cystobasidiomycetes	Erythrobasidiales	Erythrobasidiaceae	*Erythrobasidium hasegawianum*	0.022	0.003	0.011
Ascomycota	Dothideomycetes	Chaetothyriales	Herpotrichiellaceae	*Exophiala* sp.	0.003	0.000	0.001
Ascomycota	Dothideomycetes	Chaetothyriales	Herpotrichiellaceae	*Exophiala xenobiotica*	0.049	0.000	0.020
				Fungi	4.735	3.577	4.048
Ascomycota	Sordariomycetes	Hypocreales	Nectriaceae	*Fusarium solani*	0.010	0.027	0.020
Ascomycota	Sordariomycetes	Hypocreales	Nectriaceae	*Fusarium* sp.	0.148	0.022	0.073
Basidiomycota	Geminibasidiomycetes	Geminibasidiales	Geminibasidiaceae	*Geminibasidium* sp.	0.004	0.000	0.002
Ascomycota	Sordariomycetes	Hypocreales	Nectriaceae	*Gibberella intricans*	0.106	0.019	0.054
Basidiomycota	Exobasidiomycetes	Golubeviales	Golubeviaceae	*Golubevia pallescens*	0.092	0.000	0.037
Ascomycota	Sordariomycetes	Microascales	Halosphaeriaceae	Halosphaeriaceae	0.010	0.000	0.004
Basidiomycota	Tremellomycetes	Tremellales	Bulleribasidiaceae	*Hannaella oryzae*	0.000	0.006	0.004
Ascomycota	Dothideomycetes	Dothideales	*Incertae sedis*	*Hortaea werneckii*	0.122	3.205	1.951
Ascomycota	Sordariomycetes	Hypocreales		Hypocreales	0.005	0.000	0.002
Ascomycota	Sordariomycetes	Xylariales	Xylariaceae	*Hypoxylon monticulosum*	0.032	0.000	0.013
Ascomycota	Dothideomycetes	Pleosporales	Lentitheciaceae	*Keissleriella yonaguniensis*	0.409	0.003	0.168
Basidiomycota	Tremellomycetes	Tremellales	Cuniculitremaceae	*Kockovaella sacchari*	0.262	0.000	0.106
Ascomycota	Dothideomycetes	Pleosporales	Phaeosphaeriaceae	*Leptospora rubella*	0.518	0.000	0.211
Basidiomycota	Malasseziomycetes	Malasseziales	Malasseziaceae	*Malassezia restricta*	0.000	0.009	0.005
Ascomycota	Dothideomycetes	Pleosporales	Trematosphaeriaceae	*Medicopsis romeroi*	0.000	0.003	0.002
Basidiomycota	Exobasidiomycetes	Exobasidiales	Brachybasidiaceae	*Meira argovae*	0.037	0.000	0.015
Ascomycota	Dothideomycetes	Capnodiales	Mycosphaerellaceae	*Mycosphaerella etlingerae*	0.008	0.000	0.003
Ascomycota	Dothideomycetes	Capnodiales	Mycosphaerellaceae	*Mycosphaerella* sp.	0.078	0.000	0.032
Ascomycota	Dothideomycetes	Capnodiales	Mycosphaerellaceae	Mycosphaerellaceae	2.517	0.556	1.354
Ascomycota	Sordariomycetes	Hypocreales	Nectriaceae	Nectriaceae	0.010	0.000	0.004
Ascomycota	Sordariomycetes	Xylariales	Apiosporaceae	*Nigrospora oryzae*	0.240	0.051	0.128
Ascomycota	Dothideomycetes	Venturiales	Sympoventuriaceae	*Ochroconis musae*	0.009	0.000	0.004
Basidiomycota	Tremellomycetes	Tremellales	Rhynchogastremataceae	*Papiliotrema pseudoalba*	0.840	0.212	0.467
Basidiomycota	Tremellomycetes	Tremellales	Rhynchogastremataceae	*Papiliotrema* sp.	0.000	0.194	0.115
Ascomycota	Dothideomycetes	Pleosporales	Didymosphaeriaceae	*Paraconiothyrium* sp.	0.032	0.000	0.013
Ascomycota	Eurotiomycetes	Eurotiales	Aspergillaceae	*Penicillium* sp.	0.000	0.013	0.007
Basidiomycota	Agaricomycetes	Russulales	Peniophoraceae	*Peniophora* sp.	0.002	0.004	0.003
Ascomycota	Sordariomycetes	Xylariales	Sporocadaceae	*Pestalotiopsis rhododendri*	0.005	0.000	0.002
Ascomycota	Dothideomycetes	Capnodiales	Mycosphaerellaceae	*Phaeophleospora hymenocallidicola*	0.083	0.085	0.084
Ascomycota	Dothideomycetes	Pleosporales	Phaeosphaeriaceae	Phaeosphaeriaceae	0.000	0.031	0.018
Basidiomycota	Agaricomycetes	Polyporales	Meruliaceae	*Phanerochaete tuberculata*	0.028	0.024	0.025
Ascomycota	Sordariomycetes	Xylariales	*Incertae sedis*	*Phialemoniopsis ocularis*	0.007	0.000	0.003
Ascomycota	Sordariomycetes	Diaporthales	Valsaceae	*Phomopsis* sp.	0.012	0.042	0.030
Ascomycota	Dothideomycetes	Botryosphaeriales	Phyllostictaceae	*Phyllosticta capitalensis*	0.000	0.007	0.004
Ascomycota	Sordariomycetes	Glomerellales	Plectosphaerellaceae	Plectosphaerellaceae	0.014	0.000	0.006
Ascomycota	Dothideomycetes	Pleosporales	Pleosporaceae	Pleosporaceae	0.000	0.021	0.013
Ascomycota	Dothideomycetes	Pleosporales		Pleosporales sp. 1	0.141	0.037	0.080
Ascomycota	Dothideomycetes	Pleosporales		Pleosporales sp. 2	0.059	0.000	0.024
Ascomycota	Dothideomycetes	Pleosporales	Sporomiaceae	*Preussia persica*	0.091	0.082	0.085
Basidiomycota	Agaricomycetes	Agaricales	Strophariaceae	*Psilocybe coprophila*	0.003	0.000	0.001
Basidiomycota	Agaricomycetes	Agaricales	Strophariaceae	*Psilocybe* sp.	0.015	0.000	0.006
Ascomycota	Dothideomycetes	Pleosporales	Cucurbitariaceae	*Pyrenochaetopsis leptospora*	1.409	1.141	1.250
Ascomycota	Dothideomycetes	Pleosporales	Cucurbitariaceae	*Pyrenochaetopsis* sp.	1.146	0.146	0.552
Ascomycota	Dothideomycetes	Capnodiales	Dissoconiaceae	*Ramichloridium luteum*	0.048	0.000	0.020
Basidiomycota	Microbotryomycetes	Sporidiobolales	Sporidiobolaceae	*Rhodotorula mucilaginosa*	0.086	0.123	0.108
Basidiomycota	Agaricomycetes	Polyporales	Meripilaceae	*Rigidoporus* sp.	0.013	0.019	0.017
Ascomycota	Dothideomycetes	Pleosporales	Thyridariaceae	*Roussoella solani*	0.000	0.007	0.004
Basidiomycota	Tremellomycetes	Tremellales	Trimorphomycetaceae	*Saitozyma flava*	0.137	0.000	0.056
Ascomycota	Dothideomycetes	Pleosporales	Phaeosphaeriaceae	*Sclerostagonospora ericae*	0.033	0.124	0.087
Ascomycota	Dothideomycetes	Pleosporales	Phaeosphaeriaceae	*Sclerostagonospora phragmiticola*	0.169	1.525	0.973
Ascomycota	Dothideomycetes	Pleosporales	Lentitheciaceae	*Setoseptoria arundinacea*	0.123	0.000	0.050
Ascomycota	Sordariomycetes	Hypocreales	Cordycipitaceae	*Simplicillium obclavatum*	0.010	0.008	0.009
Ascomycota	Sordariomycetes	Hypocreales	Cordycipitaceae	*Simplicillium* sp.	0.001	0.000	0.001
Ascomycota	Sordariomycetes			Sordariomycetes	0.035	0.010	0.020
Basidiomycota	Microbotryomycetes	Sporidiobolales	Sporidiobolaceae	*Sporobolomyces koalae*	0.029	0.000	0.012
Ascomycota	Dothideomycetes	Pleosporales	Massarinaceae	*Stagonospora neglecta*	0.035	0.173	0.117
Ascomycota	Dothideomycetes	Pleosporales	Pleosporaceae	*Stemphylium vesicarium*	0.000	0.030	0.018
Ascomycota	Dothideomycetes	Chaetothyriales	Incertae sedis	*Strelitziana africana*	0.010	0.000	0.004
Ascomycota	Dothideomycetes	Chaetothyriales	*Incertae sedis*	*Strelitziana eucalypti*	0.013	0.000	0.005
Basidiomycota	Cystobasidiomycetes		Symmetrosporaceae	*Symmetrospora* sp.	0.067	0.029	0.044
Ascomycota	Dothideomycetes	Capnodiales	Teratosphaeriaceae	*Teratosphaeria* sp.	0.013	0.136	0.086
Ascomycota	Dothideomycetes	Capnodiales	Teratosphaeriaceae	Teratosphaeriaceae	0.798	4.551	3.024
Basidiomycota	Agaricomycetes	Polyporales	Coriolaceae	*Trametes cubensis*	0.004	0.006	0.005
Basidiomycota	Tremellomycetes	Tremellales		Tremellales	0.032	0.000	0.013
Ascomycota	Sordariomycetes	Hypocreales	Hypocreaceae	*Trichoderma lixii*	0.000	0.011	0.007
Ascomycota	Sordariomycetes	Hypocreales	Hypocreaceae	*Trichoderma* sp.	0.035	0.044	0.040
Ascomycota	Dothideomycetes	Chaetothyriales	Trichomeriaceae	Trichomeriaceae	0.049	0.000	0.020
Ascomycota	Dothideomycetes	Capnodiales	Dissoconiaceae	*Uwebraunia musae*	0.003	0.000	0.001
Ascomycota	Dothideomycetes	Chaetothyriales	Herpotrichiellaceae	*Veronaea botryosa*	0.004	0.000	0.002
Ascomycota	Dothideomycetes	Pleosporales	Sporomiaceae	*Westerdykella dispersa*	0.332	0.277	0.299
Ascomycota	Saccharomycetes	Saccharomycetales	Phaffomycetaceae	*Wickerhamomyces anomalus*	0.000	0.006	0.003
Ascomycota	Sordariomycetes	Xylariales		Xylariales	0.011	0.000	0.004
Ascomycota	Dothideomycetes	Capnodiales	Mycosphaerellaceae	*Zasmidium* sp.	0.108	0.000	0.044

At genus and species levels, taxa having the highest percentage of occurrence included *Alternaria* sp. (3.46%), *Cladosporium delicatulum* (2.56%) and *Pyrenochaetopsis leptospora* (1.41%) in the winter samples, and *Aureobasidium* sp. (10.72%), *Cladosporium* sp. (7.90%), *C. delicatulum* (3.45%) and *Hortaea werneckii* (3.21%) in the summer samples (Table 3). These latter four species also had the highest overall percentage of occurrence (both seasons).

Calculated from the read numbers of the different OTUs, the fungal community in the winter samples had a higher species richness of 7.50 (Margalef) than that in the summer samples (5.29) but the Shannon–Wiener Diversity Index was comparable between the two samples, 1.98 and 2.09 respectively (Table 2). The overall Margalef and Shannon–Wiener Diversity indices were 8.11 and 2.28, respectively. The fungal community in both winter and summer samples had reached species saturation and the winter samples had a higher species diversity (Fig. 3B).

**Figure 5:**
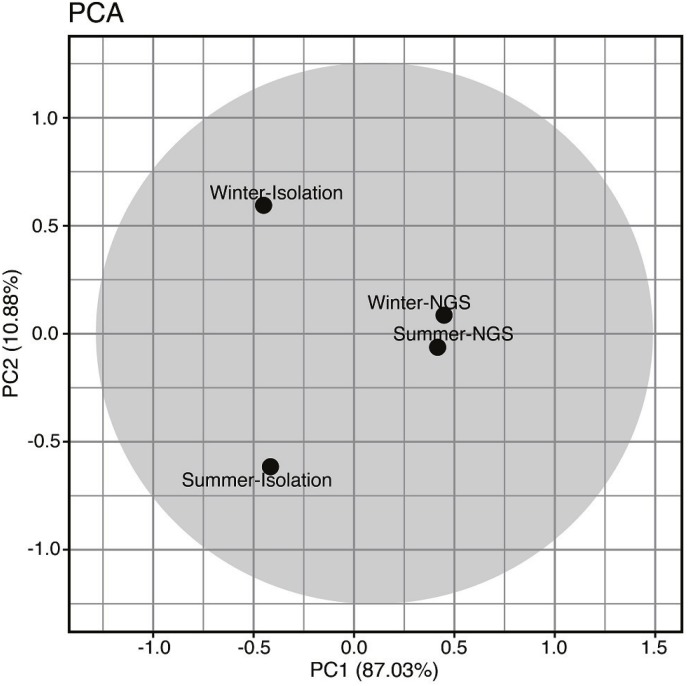
Principle component analysis based on percentage of occurrence of foliar endophytic fungal communities of *Acanthusilicifolius* var. *xiamenensis* in summer and winter seasons obtained from isolation and metabarcoding (NGS) studies.

The fungal community among isolation/metabarcoding and winter/summer samples were analyzed by PCA and the result is shown in Fig. 5. A large extent of community variation was found across PC1 (87.03%), and to a lesser extent across PC2 (10.88%). Separation across PC1 was associated with changes in fungal composition between the methods; the fungal communities obtained from the metabarcoding method (Winter-NGS, Summer-NGS) were positively correlated while those obtained from the isolation method (Winter-Isolation, Summer-Isolation) were negatively correlated. For PC2, the community variation was associated with the summer and winter samples; the winter samples (Winter-NGS, Winter-Isolation) and the summer samples (Summer-NGS, Summer Isolation) were positively and negatively correlated, respectively.

### Total diversity of fungi on *Acanthus ilicifolius* var. *xiamenensis*

Based on the average of the percentage of occurrence in the isolation study (Table 1) and metabarcoding analysis (Table 3), the phylum, class and order classifications of the fungi associated with *A. ilicifolius* var. *xiamenensis* were obtained (Figs. 4G–4I). Ascomycota was still dominant, especially in the winter samples (Fig. 4G). The dominant classes in the winter and summer samples were Dothideomycetes and Sordariomycetes (Fig. 4H). Capnodiales, Diaporthales, Glomerellales and Pleosporales were dominant orders in both seasons, although varying in percentage of occurrences (Fig. 4I). The percentages of Dothideales and Hypocreales were much higher in the summer than in the winter.

Table 4 lists the species of fungi identified from the isolation and metabarcoding methods, excluding those taxa at the family level or above. Excluding the composite taxa (i.e., spp.), *H. werneckii* and *Setoseptoria arundinacea* were the only fungi recovered from both methods and at least 110 species were identified from leaves of *A. ilicifolius* var. *xiamenensis*. The most speciose genus on *A. ilicifolius* var. *xiamenensis* was *Colletotrichum*. Some genera were only obtained with the fungal isolation procedure such as *Diaporthe* spp., *Phoma* spp. and *Pseudocercospora* spp. while some were only recovered with the metabarcoding study, such as *Aspergillus* spp., *Exophiala* spp., *Trichoderma* spp. etc. Species of *Alternaria*, *Aureobasidium*, *Cladosporium*, *Colletotrichum*, *Fusarium*, *Nigrospora*, *Pestalotiopsis* and *Phomopsis* were identified with both methods.

**Table 4:** Fungal taxa associated with leaves of *Acanthus ilicifolius* var. *xiamenensis*. The list was summarized from results of the isolation and metabarcoding analyses.

**ASCOMYCOTA**		**BASIDIOMYCOTA**
**Botryosphaeriales**	**Hypocreales**	**Agaricales**
*Aplosporella yalgorensis*	*Acremonium polychromum*	*Psilocybe coprophila*
*Botryosphaeria dothidea^a^*	*Fusarium oxysporum^a^*	*Psilocybe* sp.
*Guignardia* sp.^a^	*Fusarium solani*	**Erythrobasidiales**
*Phyllosticta capitalensis*	*Fusarium* spp.^b^	*Erythrobasidium hasegawianum*
**Capnodiales**	*Gibberella intricans*	**Exobasidiales**
*Acidiella uranophila^a^*	*Simplicillium obclavatum*	*Meira argovae*
*Cladosporium delicatulum*	*Simplicillium* sp.	**Geminibasidiales**
*Cladosporium* spp.^b^	*Trichoderma lixii*	*Geminibasidium* sp.
*Cladosporium sphaerospermum*	*Trichoderma* sp.	**Golubeviales**
*Devriesia* sp.	**Pleosporales**	*Golubevia pallescens*
*Mycosphaerella etlingerae*	*Alternaria alternata^a^*	**Hymenochaetales**
*Mycosphaerella* sp.	*Alternaria* spp.^b^	*Phellinus noxius^a^*
*Phaeophleospora eucalypticola^a^*	*Coniothyrium sidae*	**Malasseziales**
*Phaeophleospora hymenocallidicola*	*Corynespora cassiicola^a^*	*Malassezia restricta*
*Pseudocercospora nymphaeacea^a^*	*Didymella* spp.^b^	**Polyporales**
*Pseudocercospora* sp.^a^	*Drechslera dematioidea^a^*	*Bjerkandera adusta*
*Ramichloridium luteum*	*Keissleriella yonaguniensis*	*Phanerina mellea^a^*
*Ramichloridium punctatum^a^*	*Leptospora rubella*	*Phanerochaete tuberculata*
*Teratosphaeria capensis^a^*	*Medicopsis romeroi*	*Rigidoporus* sp.
*Teratosphaeria* sp.	*Paraconiothyrium* sp.	*Tinctoporellus epimiltinus^a^*
*Uwebraunia musae*	*Parastagonospora phoenicicola^a^*	**Russulales**
*Zasmidium citri^a^*	*Phoma* spp.^a^	*Peniophora* sp.
*Zasmidium* sp.	*Preussia persica*	**Sporidiobolales**
**Chaetothyriales**	*Pyrenochaetopsis leptospora*	*Rhodotorula mucilaginosa*
*Capronia semi-immersa*	*Pyrenochaetopsis* sp.	*Sporobolomyces koalae*
*Cyphellophora sessilis*	*Roussoella solani*	**Tremellales**
*Exophiala* sp.	*Sclerostagonospora ericae*	*Bullera unica*
*Exophiala xenobiotica*	*Sclerostagonospora phragmiticola*	*Cryptococcus dimennae*
*Strelitziana africana*	*Septoriella hubertusii^a^*	*Derxomyces* sp.
*Strelitziana eucalypti*	*Setoseptoria arundinacea^b^*	*Dioszegia* sp.
*Veronaea botryosa*	*Stagonospora neglecta*	*Dioszegia takashimae*
**Diaporthales**	*Stagonosporopsis cucurbitacearum^a^*	*Hannaella oryzae*
*Diaporthe endophytica^a^*	*Stemphylium vesicarium*	*Kockovaella sacchari*
*Diaporthe longicolla^a^*	*Westerdykella dispersa*	*Papiliotrema pseudoalba*
*Diaporthe phaseolorum^a^*	**Saccharomycetales**	*Papiliotrema* sp.
*Phomopsis asparagi*	*Wickerhamomyces anomalus*	*Saitozyma flava*
*Phomopsis* spp.^b^	**Venturiales**	*Trametes cubensis*
**Dothideales**	*Ochroconis musae*	**Basidiomycota order*incertae sedis***
*Aureobasidium* spp.^b^	**Xylariales**	*Symmetrospora* sp.
*Aureobasidium thailandense*	*Arthrinium* sp.	
*Hortaea werneckii^b^*	*Daldinia eschscholtzii^a^*	
**Eurotiales**	*Hypoxylon monticulosum*	
*Aspergillus penicillioides*	*Nigrospora oryzae*	
*Aspergillus* sp.	*Nigrospora* sp.^a^	
*Penicillium* sp.	*Nodulisporium* sp.^a^	
**Glomerellales**	*Pestalotiopsis microspora^a^*	
*Colletotrichum boninense^a^*	*Pestalotiopsis rhododendri*	
*Colletotrichum brasiliense*	*Phialemoniopsis ocularis*	
*Colletotrichum gloeosporioides*	*Xylaria* sp.^a^	
*Colletotrichum hippeastri^a^*		
*Colletotrichum* spp.^a^		

**Notes.**

a From isolation.

b from both methods.

## Discussion

This study investigated the diversity of fungi associated with leaves of the mangrove plant *Acanthus ilicifolius* var. *xiamenensis* using the traditional isolation technique and the metabarcoding approach. In the isolation study, most of the isolates did not fruit on the agar plates and sequence analysis of the internal transcribed spacer regions of the rDNA including the 5.8S rDNA (ITS) was used to identify the cultures. ITS is easily amplifiable by PCR and has the highest probability of successful identification for the broadest range of fungi as compared to other rDNA regions and protein genes (Schoch et al., 2012). In the metabarcoding analysis, many OTUs were only identified to the phylum or kingdom levels (Table 3) and the UNITE database was not extensive enough to identify these sequences down to genus/species level (Nilsson et al., 2019). However, the metabarcoding approach offers the advantages of finding signatures of unculturable fungi and potential cryptic species not identifiable with other methods. The nested PCR approach used in this study was found to be able to specifically amplify fungal sequences in the samples.

The leaves were surface-sterilized before isolation and therefore the diversity of fungi recovered from isolation represented the endophytic fungal diversity. On the other hand, the diversity obtained from the metabarcoding analysis represented predominantly endophytic fungi and might represent partial diversity of the epiphytic fungi as surface sterilization of leaves by sodium hypochlorite and ethanol does not completely eliminate all fungal DNA on the surface of the leaves (Burgdorf et al., 2014). This might have resulted in the differences in fungal richness (Margalef species richness, total richness) between the two methods, i.e., generally higher in the metabarcoding analysis (winter: 7.50 (Margalef), 96 species, summer: 5.29, 70) than in the isolation study (winter: 6.24, 30, summer: 5.44, 26) although the Shannon–Wiener diversity index of the two samples was comparable.

The winter samples had a higher fungal species diversity. The weather conditions of Kinmen, Taiwan in January 2014, when the winter samples were collected, were much colder and drier (13.7 °C, 0 mm rainfall, 65% relative humidity) than July 2014 (29.8 °C, 106.9 mm rainfall, 81% relative humidity) for the summer samples. Generally, higher richness and abundance of endophytic fungi were found in hotter and wetter seasons (Pang et al., 2008).

Only nine out of a total of 47 fungal species isolated from *A. ilicifolius* var. *xiamenensis* were common between the two sampling times, showing a seasonal variation of fungal diversity using the culture method. However from the metabarcoding analysis, 55 taxa were found to be common between the winter and summer samples (41 and 15 exclusive fungi in the winter and summer samples, respectively), suggesting there was an overall similarity in fungal diversity between the samples. These results show the weakness of using isolation techniques as the sole methods to study diversity of endophytic fungi of mangrove plants (Abdelfattah et al., 2015) Inoculation of leaf discs on a nutritious medium always favors fast-growing fungi to be isolated. In addition, the isolation medium (MEAF) used in this study only recovered a fraction of culturable fungal diversity and it is advisable to use multiple media to widen the number of fungal isolates (Rosa et al., 2011; Potshangbam et al., 2017). Three basidiomycetes *Phellinus noxius*, *Phanerina mellea* and *Tinctoporellus epimiltinus* were isolated from the summer samples, but a number of basidiomycetous OTUs were recovered from both seasons from the metabarcoding analysis and this further confirms the importance of culture-independent techniques in studying diversity of fungi.

A core group of culturable endophytic fungi was found to be associated with mangrove plants, including species of the genera *Acremonium*, *Cladosporium*, *Colletotrichum*, *Fusarium*, *Pestalotiopsis*, *Phyllosticta* (sexual morph *Guignardia*), *Phoma*, *Phomopsis* (sexual morph *Diaporthe*) and *Sporomiella* (Pang et al., 2008). Many of these genera, such as *Acremonium*, *Cladosporium*, *Phomopsis*, *Phyllosticta*, among others, were isolated from leaves of *A. ilicifolius* var. *xiamenensis* in this study, confirming their prevalence in mangrove plants. However, *Sporomiella*, a universal endophytic taxon of mangrove plants, was not found in this study (Pang et al., 2008). The number of species isolated from leaves of *A. ilicifolius* var. *xiamenensis* (47) was much higher than those found in related studies in this species: 11 species from roots in Udupi, India (Ananda & Sridhar, 2002), 10 species from leaves in Ranong, Thailand (Chaeprasert et al., 2010), eight species from leaves in Tamil Nadu, India (Suryanarayanan & Kumaresan, 2000) and 14 species from leaves in Muthupet, India (Priyadharshini, Ambikapathy & Panneerselvam, 2015). However, the fungal community obtained from the metabarcoding analysis was different from that of the isolation study. The dominant fungi included *Cladosporium* spp. and other common terrestrial fungi, such as *Hortaea werneckii*. *H. werneckii* is a cosmopolitan halophilic fungus and can potentially cause human diseases (Marchetta et al., 2018). Together with *Setoseptoria arundinacea*, *H. werneckii* was also cultured from leaves of *A. ilicifolius* var. *xiamenensis* and it was previously reported from surface-sterilized roots and stems of the mangrove plant *Aegiceras corniculatum* (Chen et al., 2012).

At least 110 species (excluding the composite genera) were obtained from both isolation and metabarcoding studies suggesting a much higher fungal diversity associated with leaves of *A. ilicifolius* var. *xiamenensis*. Ascomycota was dominant with a small proportion of Basidiomycota from both methods, agreeing with similar studies using the traditional culture methods (Hamzah et al., 2018; Zhou et al., 2018) and with Arfi et al. (2012) who used a culture-independent approach. As expected, Basidiomycetes were not commonly cultured as endophytes (Chaeprasert et al., 2010; Xing & Guo, 2011; Costa, Maia & Cavalcanti, 2012).

Dothideomycetes was found to be the most dominant class in both seasons from both methods. Dothideomycetes were also found to be the dominant class of fungi on the aerial parts (trunk, bark and leaf) of the mangrove plants *Avicennia marina* and *Rhizophora stylosa* and Lecanoromycetes in *R. stylosa* using 454 pyrosequencing of 18S and ITS rDNA genes (Arfi et al., 2012). Lecanoromycetes is a group of lichenized fungi; it was not found in this study, probably because tree trunk and bark, where this group of fungi normally inhabits, were not analyzed. Also according to Arfi et al. (2012), Capnodiales, Diaporthales, Dothideales and Pleosporales were dominant on the emerged plant parts, especially in *A. marina*. This result generally agrees with this study, with variations related to the abundance.

A number of fungi recovered from *A. ilicifolius* var. *xiamenensis* are well known pathogens such as *Cladosporium*, *Colletotrichum*, and *Fusarium*, which might ultimately cause plant diseases. The Botryosphaeriales was reported to potentially cause diseases of mangrove plants (Osorio et al., 2017). In this study, *Aplosporella yalgorensis*, *Botryosphaeria dothidea*, *Guignardia* sp. and *Phyllosticta capitalensis* of the Botryosphaeriales were recovered. Whether these fungi cause diseases in *A. ilicifolius* var. *xiamenensis* is not known and requires further research. A *Purpureocillium* sp. isolated endophytically from roots of *Kandelia candel* was found to protect growth of the plant from copper(II) stress when this fungus was added to the growth pots (Gong et al., 2017). Whether endophytic fungi help to relief metal stress imposed on mangrove plants also requires further studies. A high quantity of RNA transcripts of fungi from surface-sterilized leaves of *A. marina* was found (Huang et al., 2014) and it may suggest that endophytic fungi live in a close symbiotic relationship with the mangrove plant.

In conclusion, this study discovered a high diversity of fungi associated with leaves of *A. ilicifolius* var. *xiamenensis* with a total of 110 taxa recovered from the isolation and metabarcoding methods. From the isolation study, Ascomycota was dominant, with Basidiomycota isolated only in the summer samples. *C. cassiicola* (6.90%), *F. oxysporum* (6.40%) and *Guignardia* sp. (6.40%) had the highest overall percentage of occurrence. In the metabarcoding analysis, Ascomycota was also dominant over the Basidiomycota. Based on reads, *Aureobasidium* sp. (10.72%), *Cladosporium* sp. (7.90%), *C. delicatulum* (3.45%) and *H. werneckii* (3.21%) had the highest percentage of occurrence. The use of both methods discovered a much higher diversity of endophytic fungi associated with *A. ilicifolius* var. *xiamenensis*. The association of these fungi with the plant is not known and future studies should focus on the ecological roles of these fungi. However, a chemical analysis of the spent culture liquid of the fungal isolates in this study suggests that 28 isolates produced antimicrobial substances against some Gram-positive and Gram-negative bacteria and fungi and thus might protect the plant from microbial diseases (Chi et al., 2019).

##  Supplemental Information

10.7717/peerj.7293/supp-1 Supplemental Information 1 Raw sequence data for internal transcribed spacer regions of the rDNA including 5.8S (ITS) of fungi isolated from Acanthus ilicifolius var. xiamenensis The fungal isolates were cultured from leaves of Acanthus ilicifolius var. xiamenensis collected at Lieyu Township, Kinmen County, Taiwan on malt extract freshwater agar supplemented with antibiotics.

## Additional Information and Declarations

### DNA Deposition

The following information was supplied regarding the deposition of DNA sequences: All identified fungal sequences are accessible in GenBank. The accession numbers are available in Table 1.

### Data Availability

The following information was supplied regarding data availability: All relevant data are available in Tables 1–4.
