# Supplementary material for: A highly diverse fungal community associated with leaves of the mangrove plant Acanthus ilicifolius var. xiamenensis revealed by isolation and metabarcoding analyses
Source: PeerJ. 2019 Jul 9;7:e7293. doi: 10.7717/peerj.7293 (PMC6625500; doi:10.7717/peerj.7293)
Supplement: Supplemental Information 1 — The fungal isolates were cultured from leaves of Acanthus ilicifolius var. xiamenensis collected at Lieyu Township, Kinmen County, Taiwan on malt extract freshwater agar supplemented with antibiotics. [file peerj-07-7293-s001.docx]

Results of DNA sequences

**4318(MK432973)**

AACCTGCGGAAGGATCATTACTGAAATGTAATAACTTCTATTGAAAGGTTCCAGAGTAGGCGCTACAACGCCGAAATGACCTTCTCACCCTTGTGTACTCACTATGTTGCTTTGGCGGGTCGACCTGGTTCCGACCCAGGCGGCCGGCGCCCCCAGCCTTAACTGGCCAGGACGCCCGGCTAAGTGCCCGCCAGTATACAAAACTCAAGAATTCATTTTGTGAAGTCCTGATATATCATTTAATTGATTAAAACTTTCAACAACGGATCTCTTGGTTCTGGCATCGATGAAGAACGCAGCGAAATGCGATAAGTAATGTGAATTGCAGAATTCAGTGAATCATCGAATCTTTGAACGCACATTGCGCCCTCTGGTATTCCGGAGGGCATGCCTGTTCGAGCGTCATTTCAACCCTCAAGCTCTGCTTGGTATTGGGCAACGTCCGCTGCCGGACGTGCCTTGAAGACCTCGGCGACGGCGTCCTAGCCTCGAGCGTAGTAGTAAAATATCTCGCTTTGGAGTGCTGGGCGACGGCCGCCGGACAATCGACCTTCGGTCTATTTTTCCAAGGTTGACCTCGGATCAGGTAGGGATACCCGCTGAACTTAAGCAT---

**4320(MK432978)**

AACCTGCGGAGGGATCATTACCGAGTGTGGCGCTCCGGCGCCTCCCTCCAACCCCATGTCGAAACGACTCTGTTGCCTCGGGGGCGACCCGGCCTTCGGGCGTCGGGGCCCCCGGTGGACACCTTCATAACTCTTGCATCTCTTGCGTCTGAGTGATACATACAATCAATCAAAACTTTCAACAACGGATCTCTTGGTTCTGGCATCGATGAAGAACGCAGCGAAATGCGATAAGTAATGTGAATTGCAGAATTCAGTGAATCATCGAATCTTTGAACGCACATTGCGCCCCCTGGCATTCCGGGGGGCATGCCTGTTCGAGCGTCATTACACCACTCAAGCCTGGCTTGGTATTGAGCGCCGCGGCCTGCCCGCGCGCTCCAATGTCTCCGGCTGAGCCGTCCGTCTCTAAGCGTTGTGAATAGCGATCGCTTGCGAGGCCCGGGCGGTTCGACGCCGTTAAACCCCCCATTTTCTATGGTTGACCTCGGATCAGGTAGGGATACCCGCTGAACTTAAGCAT---

**4322(MK432986)**

AACCTGCGGAGGGATCATTACTGAGTGAGGGCCTCCGGGTCCGACCTCCAACCCTTTTGTGAACCAACCTGTTGCTTCGGGGGAGACCCCGCCGTTCGCGGCGGCGGCTCCCCCGGAGGCCCATTAACACTGCGTAACTGTTTGTCGGAGTCTTAATAAATCAATCAAAACTTTCAACAACGGATCTCTTGGTTCTGGCATCGATGAAGAACGCAGCGAAATGCGATAAGTAATGTGAATTGCAGAATTCAGTGAATCATCGAATCTTTGAACGCACATTGCGCCCCCTGGTATTCCGGGGGGCATGCCTGTCCGAGCGTCATTACACCCCTCAAGCCTGGCTTGGTATTGGGCGTCGCGGGCCTCGCCCGCGCGCCTTAAAGTCTCCCCGGCTGGACCGTCTGTCCCTAAAGCGTCGTGCAAACCTCGCGGTGGGATTTGGTGGGCCGGCCGTTAAATCTTTATCCAAGGTTGACCTCGGATCAGGTAGGGATACCCGCTGAACTTAAGCAT---

**4324(MK432994)**

AACCAGCGGAGGGATCATTACTGAGTTTACGCTCTATAACCCTTTGTGAACATACCTATAACTGTTGCTTCGGCGGGTAGGGTCTCCGTGACCCTCCCGGCCTCCCGCCCCCGGGCGGGTCGGCGCCCGCCGGAGGATAACCAAACTCTGATTTAACGACGTTTCTTCTGAGTGGTACAAGCAAATAATCAAAACTTTTAACAACGGATCTCTTGGTTCTGGCATCGATGAAGAACGCAGCGAAATGCGATAAGTAATGTGAATTGCAGAATTCAGTGAATCATCGAATCTTTGAACGCACATTGCGCCCGCCAGCATTCTGGCGGGCATGCCTGTTCGAGCGTCATTTCAACCCTCAAGCTCTGCTTGGTGTTGGGGCCCTACAGCTGATGTAGGCCCTCAAAGGTAGTGGCGGACCCTCCCCGGAGCCTCCTTTGCGTAGTAACTTTACGTCTCGCACTGGGATCCGGAGGGACTCTTGCCGTAAAACCCCCAATTTTCCAAAGGTTGACCTCGGATCAGGTAGGAATACCCGCTGAACTTAAGCAT---

**4326(MK448269)**

AACCAGCGGAGGGATCATTACTGAGTTACCGCTCTATAACCCTTTGTGAACATACCTCAACTGTTGCCTCGGCGGGCAGGCGGTAGCGATACCGCCTCCCCGGCGCCGTCCGCGGCGCGCCCGCCGGGGGATAACCAAACTCTGATTTAACGACGTTTCTTCTGAGTGGCACAAGCAAAACAATAATCAAAACTTTTAACAACGGATCTCTTGGTTCTGGCATCGATGAAGAACGCAGCGAAATGCGATAAGTAATGTGAATTGCAGAATTCAGTGAATCATCGAATCTTTGAACGCACATTGCGCCCGCCAGCATTCTGGCGGGCATGCCTGTTCGAGCGTCATTTCAACCCTCAAGCACTGCTTGGTGTTGGGGCCCTACGGCCGACGTAGGCCCCCAAAGACAGTGGCGGACCCTCCCGGAGCCTCCTTTGCGTAGTAACATTACCACCTCGCACTGGGATCCGGAGGGACTCTCCTGCCGTAAAAACCCCCCAAAAACATCACAAGGTTGACCTCGGATCAGGTAGGAATACCCGCTGAACTTAAGCAT---

**4328(MK448257)**

AACCTGCGGAAGGATCATTAAGAATCATGGATGTCGGCCACCCCCGGGGGGTCTCCACGGTCGACGTGGGGGGTCCTCGGATCGGTGCTCAGCCGTCGCTCTTCACCTTGATTATGCGCACCTTTTGTTGTTTCCTCGGCAGGTTTCCTGCCGCTGGAACCCCACAAACCCCTTGTATATAAGCATTAAAACTCTGAAAACAACGTAATTATTTACAACTTTCAACAATGGATCTCTTGGTTCTGGCATCGATGAAGAACGCAGCGAAATGCGATAAGTAGTGTGAATTGCAGAATTCAGTGAATCATCGAATCTTTGAACGCACATTGCGCCCCTTGGTACTCCATGGGGCATGCCTGTTCGAGCGTCATCTAAACCCTCAAGCCTTTTGGGTGTTGTCCCGCCTTCGCGCGGTACTCCGCCCGGTTTGGTGTTGGGCGTCTGTCCCGCCGTGCGCGCGGACTCGCCCCAAATCCATTGGCAGCGGCCCTTCAGCTTCTTGCGCAGCACAATGCGTCTCGGGGCGTAGCAAGGGCACGCGTCCACTAAGCAACCCACAGTTTGACCTCGGATCAGGTAGGGATACCCGCTGAACTTAAGCAT---

**4330 (MK432953)**

CCTGCGGAGGGATCATTACACAAATATGAAGGCGGGCTGGAACCTCTCGGGGTTACAGCCTTGCTGAATTATTCACCCTTGTCTTTTGCGTACTTCTTGTTTCCTTGGTGGGTTCGCCCACCACTAGGACAAACATAAACCTTTTGTAATTGCAATCAGCGTCAGTAACAAATTAATAATTACAACTTTCAACAACGGATCTCTTGGTTCTGGCATCGATGAAGAACGCAGCGAAATGCGATAAGTAGTGTGAATTGCAGAATTCAGTGAATCATCGAATCTTTGAACGCACATTGCGCCCTTTGGTATTCCAAAGGGCATGCCTGTTCGAGCGTCATTTGTACCCTCAAGCTTTGCTTGGTGTTGGGCGTCTTGTCTCTAGCTTTGCTGGAGACTCGCCTTAAAGTAATTGGCAGCCGGCCTACTGGTTTCGGAGCGCAGCACAAGTCGCACTCTCTATCAGCAAAGGTCTAGCATCCATTAAG---

**4332(MK432974)**

TGTAATAACTTCTATTGAAAGGTTCCAGAGTAGGCGCTACAACGCCGAAATGACCTTCTCACCCTTGTGTACTCACTATGTTGCTTTGGCGGGTCGACCTGGTTCCGACCCAGGCGGCCGGCGCCCCCAGCCTTAACTGGCCAGGACGCCCGGCTAAGTGCCCGCCAGTATACAAAACTCAAGAATTCATTTTGTGAAGTCCTGATATATCATTTAATTGATTAAAACTTTCAACAACGGATCTCTTGGTTCTGGCATCGATGAAGAACGCAGCGAAATGCGATAAGTAATGTGAATTGCAGAATTCAGTGAATCATCGAATCTTTGAACGCACATTGCGCCCTCTGGTATTCCGGAGGGCATGCCTGTTCGAGCGTCATTTCAACCCTCAAGCTCTGCTTGGTATTGGGCAACGTCCGCTGCCGGACGGGCCTTGAAGACCTCGGCGACGGCGTCCTAGCCTCGAGCGTAGTAGTAAAATATCTCGCTTTGGAGTGCTGGGCGACGGCCGCCGGACAATCGACCTTCGGTCTATTTTTCCAAGGTTGACCTCGGATCAGGTAGGGATACCCGCTGAACTTAAGCAT---

**4334(MK432967)**

AACCTGCGGAAGGATCATTACCTAGAGTTGCGGGCTTTGCCTGCCATCTCTTACCCATGTCTTTTGAGTACCTTCGTTTCCTCGGCGGGTTCGCCCGCCGGTTGGACAACACTTAAACCCTTTGTAATTGAAATCAGCGTCTGAAAAAACTTTAATAGTTACAACTTTCAACAACGGATCTCTTGGTTCTGGCATCGATGAAGAACGCAGCGAAATGCGATAAGTAGTGTGAATTGCAGAATTCAGTGAATCATCGAATCTTTGAACGCACATTGCGCCCCTTGGTATTCCATGGGGCATGCCTGTTCGAGCGTCATTTGTACCTTCAAGCTCTGCTTGGTGTTGGGTGTTTGTCTCGCCTCTGCGCGCAGACTCGCCTCAAAGCAATTGGCAGCCGGCGTATTGATTTCGGAGCGCAGTACATCTCGCGCTTTGCACTCATAACGACGACGTCCAAAAAGTACATTTTTTACACTCTTGACCTCGGATCAGGTAGGGATACCCGCTGAACTTAAGCAT---

**4336 (MK448263)**

AACCTGCGGAGGGATCATTACACAAATATGAAGGCGGGCTGGAATCTCTCGGGGTTACAGCCTTGCTGAATTATTCACCCTTGTCTTTTGCGTACTTCTTGTTTCCTTGGTGGGTTCGCCCACCACTAGGACAAACATAAACCTTTTGTAATTGCAATCAGCGTCAGTAACAAATTAATAATTACAACTTTCAACAACGGATCTCTTGGTTCTGGCATCGATGAAGAACGCAGCGAAATGCGATAAGTAGTGTGAATTGCAGAATTCAGTGAATCATCGAATCTTTGAACGCACATTGCGCCCTTTGGTATTCCAAAGGGCATGCCTGTTCGAGCGTCATTTGTACCCTCAAGCTTTGCTTGGTGTTGGGCGTCTTGTCTCTAGCTTTGCTGGAGACTCGCCTTAAAGTAATTGGCAGCCGGCCTACTGGTTTCGGAGCGCAGCACAAGTCGCACTCTCTATCAGCAAAGGTCTAGCATCCATTAAGCCTTTTTTCAACTTTTGACCTCGGATCAGGTAGGGATACCCGCTGAACTTAAGCAT---

**4338(MK432990)**

AACCTGCGGAAGGATCATTACCTAGAGTTGCGGGCTTTGCCTGCCATCTCTTACCCATGTCTTTTGAGTACCTTCGTTTCCTCGGCGGGTTCGCCCGCCGGTTGGACAACACTTAAACCCTTTGTAATTGAAATCAGCGTCTGAAAAAACTTTAATAGTTACAACTTTCAACAACGGATCTCTTGGTTCTGGCATCGATGAAGAACGCAGCGAAATGCGATAAGTAGTGTGAATTGCAGAATTCAGTGAATCATCGAATCTTTGAACGCACATTGCGCCCCTTGGTATTCCATGGGGCATGCCTGTTCGAGCGTCATTTGTACCTTCAAGCTCTGCTTGGTGTTGGGTGTTTGTCTCGCCTCTGCGCGCAGACTCGCCTCAAAGCAATTGGCAGCCGGCGTATTGATTTCGGAGCGCAGTACATCTCGCGCTTTGCACTCATAACGACGACGTCCAAAAAGTACA---

**4340 (MK448266)**

AACCTGCGGAGGGATCATTACCAGTGACCCCCGGCTTCGGCCGGGGATGATCATAACCCTTTGCTGTCCGACTCTGTTGCCTCCGGGGCGACCCTGCCTCCGGGCGGGGGCCCCGGGTGGACACATTCAAACTCTTGCGTAACTTTGCAGTCTGAGTAAATTTAATTAATAAATTAAAACTTTCAACAACGGATCTCTTGGTTCTGGCATCGATGAAGAACGCAGCGAAATGCGATAAGTAATGTGAATTGCAGAATTCAGTGAATCATCGAATCTTTGAACGCACATTGCGCCCCCTGGTATTCCGGGGGGCATGCCTGTTCGAGCGTCATTTCACCACTCAAGCCTCGCTTGGTATTGGGCGACGCGGTCCGCCGCGCGCCTCAAATCGACCGGCTGGGTCTTCTGTCCCCTCAGCGTTGTGGAAACTATTCGCTAAAGGGTGCCACGGGAGGTCACGCCGTAAAACAAGCCCATTTCTAAGGTTGACCTCGGATCAGGTAGGGATACCCGCTGAACTTAAGCAT---

**4344(MK432987)**

AACCTGCGGAGGGATCATTACTGAGTGAGGGCCTCCGGGTCCGACCTCCAACCCTTTTGTGAACCAACCTGTTGCTTCGGGGGAGACCCCGCCGTTCGCGGCGGCGGCTCCCCCGGAGGCCCATTAACACTGCGTAACTGTTTGTCGGAGTCTTAATAAATCAATCAAAACTTTCAACAACGGATCTCTTGGTTCTGGCATCGATGAAGAACGCAGCGAAATGCGATAAGTAATGTGAATTGCAGAATTCAGTGAATCATCGAATCTTTGAACGCACATTGCGCCCCCTGGTATTCCGGGGGGCATGCCTGTCCGAGCGTCATTACACCCCTCAAGCCTGGCTTGGTATTGGGCGTCGCGGGCCTCGCCCGCGCGCCTTAAAGTCTCCCCGGCTGGACCGTCTGTCCCTAAAGCGTCGTGCAAACCTCGCGGTGGGATTTGGTGGGCCGGCCGTTAAATC---

**4346(MK432960)**

AACCTGCGGAAGGATCATTATCGTAGGGGCCTCGCCCCCTTCGAGATAGCACCCTTTGTTTATGAGCACCTCTCGTTTCCTCGGCAGGCTCGCCTGCCAACGGGGACCCACCACAAACCCATTGCAGTACAAGAAGTACACGTCTGAACAAAACAAAACAAACTATTTACAACTTTCAACAACGGATCTCTTGGTTCTGGCATCGATGAAGAACGCAGCGAAATGCGATAAGTAGTGTGAATTGCAGAATTCAGTGAATCATCGAATCTTTGAACGCACATTGCGCCCTTTGGTATTCCTTAGGGCATGCCTGTTCGAGCGTCATTTCAACCCTCAAGCCTAGCTTGGTGTTGGGCGTCTGTCCCGCCTCCGCGCGCCTGGACTCGCCTCAAAAGCATTGGCGGCCGGTTCCCAGCAGGCCACGAGCGCAGCAGAGCAAGCGCTGAAGTGGCTGCGGGTCGGCACACCATGAGCCCCCCCACACCAGAATTTTGACCTCGGATCAGGTAGGGATACCCGCTGAACTTAAGCAT---

**4348(MK448249)**

AACCAGCGGAGGGATCATTACAGAGTTATCCAACTCCCAAACCCATGTGAACATATCTCTTTGTTGCCTCGGCGCAAGCTACCCGGGACCTCGCGCCCCGGGCGGCCCGCCGGCGGACAAACCAAACTCTGTTATCTTCGTTGATTATCTGAGTGTCTTATTTAATAAGTCAAAACTTTCAACAACGGATCTCTTGGTTCTGGCATCGATGAAGAACGCAGCGAAATGCGATAAGTAATGTGAATTGCAGAATTCAGTGAATCATCGAATCTTTGAACGCACATTGCGCCCATTAGTATTCTAGTGGGCATGCCTGTTCGAGCGTCATTTCAACCCCTAAGCACAGCTTATTGTTGGGCGTCTACGTCTGTAGTGCCTCAAAGACATTGGCGGAGCGGCAGCAGTCCTCTGAGCGTAGTAATTCTTTATCTCGCTTCTGTTAGGCGCTGCCCCCCCGGCCGTAAAACCCCCAATTTTTTCTGGTTGACCTCGGATCAGGTAGGAATACCCGCTGAACTTAAGCAT---

**4350 (MK448264)**

AACCTGCGGAAGGATCATTAAAGAGTAAGGGTGCTCAGCGCCCGACCTCCAACCCTTTGTTGTTAAAACTACCTTGTTGCTTTGGCGGGACCGCTCGGTCTCGAGCCGCTGGGGATTCGTCCCAGGCGAGCGCCCGCCAGAGTTAAACCAAACTCTTGTTATTAAACCGGTCGTCTGAGTTAAAATTTTGAATAAATCAAAACTTTCAACAACGGATCTCTTGGTTCTCGCATCGATGAAGAACGCAGCGAAATGCGATAAGTAATGTGAATTGCAGAATTCAGTGAATCATCGAATCTTTGAACGCACATTGCGCCCCTTGGTATTCCGAGGGGCATGCCTGTTCGAGCGTCATTACACCACTCAAGCTATGCTTGGTATTGGGTGCCGTCCTTAGTTGGGCGCGCCTTAAAGACCTCGGCGAGGCCTCACCGGCTTTAGGCGTAGTAGAATTTATTCGAACGTCTGTCAAAGGAGAGGACTTCTGCCGACTGAAACCTTTTATTTTTCTAGGTTGACCTCGGATCAGGTAGGGATACCCGCTGAACTTAAGCAT---

**4352(MK432958)**

AACCTGCGGAGGGATCATTACAAGTGACCCCGGTTTACCACCGGGATGTTCATAACCCTTTGTTGTCCGACTCTGTTGCCTCCGGGGCGACCCTGCCTTCGGGCGGGGGCTCCGGGTGGACACTTCAAACTCTTGCGTAACTTTGCAGTCTGAGTAAACTTAATTAATAAATTAAAACTTTTAACAACGGATCTCTTGGTTCTGGCATCGATGAAGAACGCAGCGAAATGCGATAAGTAATGTGAATTGCAGAATTCAGTGAATCATCGAATCTTTGAACGCACATTGCGCCCCCTGGTATTCCGGGGGGCATGCCTGTTCGAGCGTCATTTCACCACTCAAGCCTCGCTTGGTATTGGGCAACGCGGTCCGCCGCGTGCCTCAAATCGACCGGCTGGGTCTTCTGTCCCCTAAGCGTTGTGGAAACTATTCGCTAAAGGGTGCTCGGGAGGCTACGCCGTAAAACAACCCCATTTCTAAGGTTGACCTCGGATCAGGTAGGGATACCCGCTGAACTTAAGCAT---

**4354(MK432968)**

AACCTGCGGAAGGATCATTACCTAGAGTTGCGGGCTTTGCCTGCCATCTCTTACCCATGTCTTTTGAGTACCTTCGTTTCCTCGGCGGGTTCGCCCGCCGGTTGGACAACACTTAAACCCTTTGTAATTGAAATCAGCGTCTGAAAAAACTTTAATAGTTACAACTTTCAACAACGGATCTCTTGGTTCTGGCATCGATGAAGAACGCAGCGAAATGCGATAAGTAGTGTGAATTGCAGAATTCAGTGAATCATCGAATCTTTGAACGCACATTGCGCCCCTTGGTATTCCATGGGGCATGCCTGTTCGAGCGTCATTTGTACCTTCAAGCTCTGCTTGGTGTTGGGTGTTTGTCTCGCCTCTGCGCGCAGACTCGCCTCAAAGCAATTGGCAGCCGGCGTATTGATTTCGGAGCGCAGTACATCTCGCGCTTTGCACTCATAACGACGACGTCCAAAAAGTACATTTTTTACACTCTTGACCTCGGATCAGGTAGGGATACCCGCTGAACTTAAGCAT---

**4356(MK432995)**

AACCAGCGGAGGGATCATTACTGAGTTTACGCTCTATAACCCTTTGTGAACATACCTATAACTGTTGCTTCGGCGGGTAGGGTCTCCGTGACCCTCCCGGCCTCCCGCCCCCGGGCGGGTCGGCGCCCGCCGGAGGATAACCAAACTCTGATTTAACGACGTTTCTTCTGAGTGGTACAAGCAAATAATCAAAACTTTTAACAACGGATCTCTTGGTTCTGGCATCGATGAAGAACGCAGCGAAATGCGATAAGTAATGTGAATTGCAGAATTCAGTGAATCATCGAATCTTTGAACGCACATTGCGCCCGCCAGCATTCTGGCGGGCATGCCTGTTCGAGCGTCATTTCAACCCTCAAGCTCTGCTTGGTGTTGGGGCCCTACAGCTGATGTAGGCCCTCAAAGGTAGTGGCGGACCCTCCCGGAGCCTCCTTTGCGTAGTAACTTTACGTCTCGCACTGGGATCCGGAGGGACTCTTGCCGTAAAACCCCCAATTTTCCAAAGGTTGACCTCGGATCAGGTAGGAATACCCGCTGAACTTAAGCAT---

**4358(MK448267)**

AACCAGCGGAGGGATCATTACTGAGTTACCGCTCTATAACCCTTTGTGAACATACCTACAACTGTTGCTTCGGCGGGTAGGCCGTCCCGTGAAAAGGACGCCTCCCGGCCCGGCCCGGACCCCCCGCGGGAACGGACCCGGCGCCCGCCGGAGGATAACCAAACTCTGTTTTAACGACGTTTCTTCTGAGTGGCATAAGCAAAATAATCAAAACTTTTAACAACGGATCTCTTGGTTCTGGCATCGATGAAGAACGCAGCGAAATGCGATACGTAATGCGAATTGCAGAATTCAGTGAGTCATCGAATCTTTGAACGCACATTGCGCCCGCCAGCATTCTGGCGGGCATGCCTGTTCGAGCGTCATTTCAACCCTCAAGCTCTGCTTGGCGTTGGGGCTCTACGGTTGACGTAGGCCCCCAAAGGTAGTGGCGGACCCTCCCGGAGCCTCCTTTGCGTAGTAACATTTCGTCTCGCACTGGGATCCGGAGGGACTCTTGCCGTAAAACCCCCCAATTTTTCAAGGTTGACCTCGGATCAGGTAGGAATACCCGCTGAACTTAAGCAT---

**4360(MK448258)**

AACCTGCGGAAGGATCATTACCTAGAGTTGCGGGCTTTGCCTGCCATCTCTTACCCATGTCTTTTGAGTACCTTCGTTTCCTCGGCGGGTTCGCCCGCCGGTTGGACAACACTTAAACCCTTTGTAATTGAAATCAGCGTCTGAAAAACTTTAATAGTTACAACTTTCAACAACGGATCTCTTGGTTCTGGCATCGATGAAGAACGCAGCGAAATGCGATAAGTAGTGTGAATTGCAGAATTCAGTGAATCATCGAATCTTTGAACGCACATTGCGCCCCTTGGTATTCCATGGGGCATGCCTGTTCGAGCGTCATTTGTACCTTCAAGCTCTGCTTGGTGTTGGGTGTTTGTCTCGCCTTTTGCGCGCAGACTCGCCTCAAAACGATTGGCAGCCGGCGTATTGATTTCGGAGCGCAGTACATCTCGCGCTTTGCACTCACAACGACGACGTCCAAAAAGTACATTTTTACACTCTTGACCTCGGATCAGGTAGGGATACCCGCTGAACTTAAGCAT---

**4362 (MK432961)**

ATCGTAGGGGCCTCGCCCCCTTCGAGATAGCACCCTTTGTTTATGAGCACCTCTCGTTTCCTCGGCAGGCTCGCCTGCCAACGGGGACCCACCACAAACCCATTGCAGTACAAGAAGTACACGTCTGAACAAAACAAAACAAACTATTTACAACTTTCAACAACGGATCTCTTGGTTCTGGCATCGATGAAGAACGCAGCGAAATGCGATAAGTAGTGTGAATTGCAGAATTCAGTGAATCATCGAATCTTTGAACGCACATTGCGCCCTTTGGTATTCCTTAGGGCATGCCTGTTCGAGCGTCATTTCAACCCTCAAGCCTAGCTTGGTGTTGGGCGTCTGTCCCGCCTCCGCGCGCCTGGACTCGCCTCAAAAGCATTGGCGGCCGGTTCCCAGCAGGCCACGAGCGCAGCAGAGCAAGCGCTGAAGTGGCTGCGGGTCGGCACACCATGAGCCCCCCCACACCAGAATTTTGACCTCGGATCAGGTAGGGATACCCGCTGAACTTAAGCAT---

**4364 (MK432989)**

CATTACTGAGTTTACGCTCTACAACCCTTTGTGAACATACCTATAACTGTTGCTTCGGCGGGTAGGGTCTCCGTGACCCTCCCGGCCTCCCGCCCCCGGGCGGGTCGGCGCCCGCCGGAGGATAACCAAACTCTGATTTAACGACGTTTCTTCTGAGTGGTACAAGCAAATAATCAAAACTTTTAACAACGGATCTCTTGGTTCTGGCATCGATGAAGAACGCAGCGAAATGCGATAAGTAATGTGAATTGCAGAATTCAGTGAATCATCGAATCTTTGAACGCACATTGCGCCCGCCAGCATTCTGGCGGGCATGCCTGTTCGAGCGTCATTTCAACCCTCAAGCTCTGCTTGGTGTTGGGGCCCTACAGCTGATGTAGGCCCTCAAAGGTAGTGGCGGACCCTCCCGGAGCCTCCTTTGCGTAGTAACTTTACGTCTCGCACTGGGATCCGGAGGGACTCTTGCCGTAAAACCCCCAATTTTCCAAAGGTTGACCTCGGATCAGGTAGGAATACCCGCTGAACTTAAGCAT---

**4366(MK432991)**

AACCTGCGGAAGGATCATTACCTAGAGTTGCGGGCTTTGCCTGCCATCTCTTACCCATGTCTTTTGAGTACCTTCGTTTCCTCGGCGGGTTCGCCCGCCGGTTGGACAACACTTAAACCCTTTGTAATTGAAATCAGCGTCTGAAAAAACTTTAATAGTTACAACTTTCAACAACGGATCTCTTGGTTCTGGCATCGATGAAGAACGCAGCGAAATGCGATAAGTAGTGTGAATTGCAGAATTCAGTGAATCATCGAATCTTTGAACGCACATTGCGCCCCTTGGTATTCCATGGGGCATGCCTGTTCGAGCGTCATTTGTACCTTCAAGCTCTGCTTGGTGTTGGGTGTTTGTCTCGCCTCTGCGCGCAGACTCGCCTCAAAGCAATTGGCAGCCGGCGTATTGATTTCGGAGCGCAGTACATCTCGCGCTTTGCACTCATAACGACGACGTCCAAAAAGTACA---

**4368 (same colony morphology as 4336)**

AACCTGCGGAGGGATCATTACACAAATATGAAGGCGGGCTGGAATCTCTCGGGGTTACAGCCTTGCTGAATTATTCACCCTTGTCTTTTGCGTACTTCTTGTTTCCTTGGTGGGTTCGCCCACCACTAGGACAAACATAAACCTTTTGTAATTGCAATCAGCGTCAGTAACAAATTAATAATTACAACTTTCAACAACGGATCTCTTGGTTCTGGCATCGATGAAGAACGCAGCGAAATGCGATAAGTAGTGTGAATTGCAGAATTCAGTGAATCATCGAATCTTTGAACGCACATTGCGCCCTTTGGTATTCCAAAGGGCATGCCTGTTCGAGCGTCATTTGTACCCTCAAGCTTTGCTTGGTGTTGGGCGTCTTGTCTCTAGCTTTGCTGGAGACTCGCCTTAAAGTAATTGGCAGCCGGCCTACTGGTTTCGGAGCGCAGCACAAGTCGCACTCTCTATCAGCAAAGGTCTAGCATCCATTAAGCCTTTTTTCAACTTTTGACCTCGGATCAGGTAGGGATACCCGCTGAACTTAAGCAT---

**4370(MK432992)**

AACCAGCGGAGGGATCATTACTGAGTTACCGCTCTATAACCCTTTGTGAACATACCTACAACTGTTGCTTCGGCGGGTAGGCCGTCCCCTGAAAAGGACGCCTCCCGGCCCGGACCGGACCCCCTGTGGGGCCGGACTCGGCGCCCGCCGGAGGATAACCAAACGCTATTTTAACGACGTTTCTTCTGAGTGGCATAAGCAAAATAATCAAAACTTTTAACAACGGATCTCTTGGTTCTGGCATCGATGAAGAACGCAGCGAAATGCGATAAGTAATGTGAATTGCAGAATTCAGTGAATCATCGAATCTTTGAACGCACATTGCGCCCGCCAGCATTCTGGCGGGCATGCCTGTTCGAGCGTCATTTCAACCCTCAAGCTCTGCTTGGTGTTGGGGCTCTACGGTCGACGTAGGCCCTCAAAGGTAGTGGCGGACCCTCCCGGAGCCTCCTTTGCGTAGTAACATTTCGTCTCGCACTGGGATCCGGAGGGACTCTTGCCGTAAAACCCCCCAATTTTTCAAGGTTGACCTCGGATCAGGTAGGAATACCCGCTGAACTTAAGCAT---

**4372(MK448279)**

AACCAGCGGAGGGATCATTACTGAGTTACCGCTCTATAACCCTTTGTGAACATACCTACAACTGTTGCTTCGGCGGGTAGGCCGTCCCGTGAAAAGGACGCCTCCCGGCCCGGCCCGGACCCCCCGCGGGAACGGACCCGGCGCCCGCCGGAGGATAACCAAACTCTGTTTTAACGACGTTTCTTCTGAGTGGCATAAGCAAAATAATCAAAACTTTTAACAACGGATCTCTTGGTTCTGGCATCGATGAAGAACGCAGCGAAATGCGATACGTAATGCGAATTGCAGAATTCAGTGAGTCATCGAATCTTTGAACGCACATTGCGCCCGCCAGCATTCTGGCGGGCATGCCTGTTCGAGCGTCATTTCAACCCTCAAGCTCTGCTTGGCGTTGGGGCTCTACGGTTGACGTAGGCCCCCAAAGGTAGTGGCGGACCCTCCCGGAGCCTCCTTTGCGTAGTAACATTTCGTCTCGCACTGGGATCCGGAGGG---

**4374(MK448254)**

AACCTGCGGAGGGATCATTACTGAGTGAGGGCTCACGCCCGACCTCCAACCCTTTGTGAACCAAACTTGTTGCTTCGGGGGCGACCCTGCCGACGACTCCGTCGCCGGGCGCCCCCGGAGGTCTTCTAAACACTGCATCTTTGCGTCGGAGTTTAAACAAATTAAACAAAACTTTCAACAACGGATCTCTTGGTTCTGGCATCGATGAAGAACGCAGCGAAATGCGATAAGTAATGTGAATTGCAGAATTCAGTGAATCATCGAATCTTTGAACGCACATTGCGCCCTTTGGTATTCCGAAGGGCATGCCTGTTCGAGCGTCATTTCACCACTCAAGCCTGGCTTGGTATTGGGCGTCGCGGTGTTTCCGCGCGCCTTAAAGTCTTCCGGCTGAGCTGTCCGTCTCTAAGCGTTGTGGATTTTTCAATTCGCTTCGGAGTGCGGGCGGCCGCGGCCGTTAAATCTTTATTCAAAGGTTGACCTCGGATCAGGTAGGGATACCCGCTGAACTTAAGCAT---

**4376(MK448273)**

AACCAGCGGAGGGATCATTGCTGGAACGCGCTTCGGCGCACCCAGAAACCCTTTGTGAACTTATACCTACTGTTGCCTCGGCGCAGGCCGGCTTCTTCACTGAGGCCCCCTGGAGACAGGGAGCAGCCCGCCGGCGGCCAACTAAACTCTTGTTTCTTAGTGAATCTCTGAGTAAAAACATAAATGAATCAAAACTTTCAACAACGGATCTCTTGGTTCTGGCATCGATGAAGAACGCAGCGAAATGCGATAAGTAATGTGAATTGCAGAATTCAGTGAATCATCGAATCTTTGAACGCACATTGCGCCCTCTGGTATTCCGGAGGGCATGCCTGTTCGAGCGTCATTTCAACCCTCAAGCCTGGCTTGGTGATGGGGCACTGCTCTCTAGCGGGAGCAGGCCCTGAAATCTAGTGGCGAGCTCGCCAGGACCCCGAGCGTAGTAGTTATATCTCGTTCTGGAAGGCCCTGGCGGTGCCCTGCCGTTAAACCCCCAACTTCTGAAAATTTGACCTCGGATCAGGTAGGAATACCCGCTGAACTTAAGCAT---

**4378(MK448281)**

TTACTGAGTTACCGCTCTATAACCCTTTGTGAACATACCTCAACTGTTGCCTCGGCGGGCAGGCGGTAGCGATACCGCCTCCCCGGCGCCGTCCGCGGCGCGCCCGCCGGGGGATAACCAAACTCTGATTTAACGACGTTTCTTCTGAGTGGCACAAGCAAAACAATAATCAAAACTTTTAACAACGGATCTCTTGGTTCTGGCATCGATGAAGAACGCAGCGAAATGCGATAAGTAATGTGAATTGCAGAATTCAGTGAATCATCGAATCTTTGAACGCACATTGCGCCCGCCAGCATTCTGGCGGGCATGCCTGTTCGAGCGTCATTTCAACCCTCAAGCACTGCTTGGTGTTGGGGCCCTACGGCCGACGTAGGCCCCCAAAGACAGTGGCGGACCCTCCCGGAGCCTCCTTTGCGTAGTAACATTACCACCTCGCACTGGGATCCGGAGGGACTCTCCTGCCGTAAAAACCCCCCAAAAACATCACAAGGTTGACCTCGGATCAGGTAGGAATACCCGCTGAACTTAAGCAT---

**4380(MK432964)**

AACCAGCGGAGGGATCATTGCTGGAACGCGCTTCGGCGCACCCAGAAACCCTTTGTGAACTTATACCTATTGTTGCCTCGGCGTAGGCCGGCCTCTTCACTGAGGCCCCCTGGAAACAGGGAGCAGCCCGCCGGCGGCCAACCAAACTCTTGTTTCTACAGTGAATCTCTGAGTAAAAAACATAAATGAATCAAAACTTTCAACAACGGATCTCTTGGTTCTGGCATCGATGAAGAACGCAGCGAAATGCGATAAGTAATGTGAATTGCAGAATTCAGTGAATCATCGAATCTTTGAACGCACATTGCGCCCTCTGGTATTCCGGAGGGCATGCCTGTTCGAGCGTCATTTCAACCCTCAAGCCTGGCTTGGTGATGGGGCACTGCCTGTAAAAGGGCAGGCCCTGAAATCTAGTGGCGAGCTCGCCAGGACCCCGAGCGTAGTAGTTATATCTCGCTCTGGAAGGCCCTGGCGGTGCCCTGCCGTTAAACCCCCAACTTCTGAAAATTTGACCTCGGATCAGGTAGGAATACCCGCTGAACTTAAGCAT---

**4382(MK448253)**

AACCAGCGGAGGGATCATTGCTGGAACGCGCCCCAGGCGCACCCAGAAACCCTTTGTGAACTTATACCTTACTGTTGCCTCGGCGCTGCTGGTCTTCACAGGCCCTTTGCTTCACAGCAAAGAGACGGCACGCCGGCGGCCAAGTTAACTATGTTTTTACACTGAAACTCTGAGAAAAAAACACAAATGAATCAAAACTTTCAACAACGGATCTCTTGGTTCTGGCATCGATGAAGAACGCAGCGAAATGCGATAAGTAATGTGAATTGCAGAATTCAGTGAATCATCGAATCTTTGAACGCACATTGCGCCCTCTGGTATTCCGGAGGGCATGCCTGTTCGAGCGTCATTTCAACCCTCAAGCACTGCTTGGTGTTGGGGCACTGCTTTTAACGAAGCAGGCCCTGAAATCTAGTGGCGAGCTCGCCAGGACCCCGAGCGCAGTAGTTAAACCCTCGCTCTGGAAGGCCCTGGCGGTGCCCTGCCGTTAAACCCCCAACTTTTGAAAATTTGACCTCGGATCAGGTAGGAATACCCGCTGAACTTAAGCAT---

**4384(MK448252)**

AACCAGCGGAGGGATCATTGCTGGAACGCGCTTCGGCGCACCCAGAAACCCTTTGTGAACTAATACCTATTGTTGCCTCGGCGCAGGCCGGCCTCTTCACTGAGGCCCCCTGGAAACAGGGAGCAGCCCGCCGGCGGCCAACCAAACTCTTGTTTCTATAGTGAATCTCTGAGTAAAAAAACATAAATGAATCAAAACTTTCAACAACGGATCTCTTGGTTCTGGCATCGATGAAGAACGCAGCGAAATGCGATAAGTAATGTGAATTGCAGAATTCAGTGAATCATCGAATCTTTGAACGCACATTGCGCCCTCTGGTATTCCGGAGGGCATGCCTGTTCGAGCGTCATTTCAACCCTCAAGCCTGGCTTGGTGATGGGGCACTGCCTGTAAAAGGGCAGGCCCTGAAATCTAGTGGCGAGCTCGCCAGGACCCCGAGCGTAGTAGTTATATCTCGCTCTGGAAGGCCCTGGCGGTGCCCTGCCGTTAAACCCCCAACTTCTGAAAATTTGACCTCGGATCAGGTAGGAATACCCGCTGAACTTAAGCAT---

**4386 (MK448280)**

AACCAGCGGAGGGATCATTACTGAGTTACCGCTCTATAACCCTTTCTGAACATACCTACAACTGTTGCTTCGGCGGGTAGGCCGTCCCGTGAAAAGGACGCCTCCCGGCCCGGCCCGGACCCCCCGCGGGAACGGACCCGGCGCCCGCCGGAGGATAACCAAACTCTGTTTTAACGACGTTTCTTCTGAGTGGCATAAGCAAAATAATCAAAACTTTTAACAACGGATCTCTTGGTTCTGGCATCGATGAAGAACGCAGCGAAATGCGATACGTAATGCGAATTGCAGAATTCAGTGAGTCATCGAATCTTTGAACGCACATTGCGCCCGCCAGCATTCTGGCGGGCATGCCTGTTCGAGCGTCATTTCAACCCTCAAGCTCTGCTTGGCGTTGGGGCTCTACGGTTGACGTAGGCCCCCAAAGGTAGTGGCGGACCCTCCCGGAGCCTCCTTTGCGTAGTAACATTTCGTCTC---

**4388(MK432975)**

AACCTGCGGAAGGATCATTACTGAAATGTAACAACTTCTATTGAAAGGTTCCAGAGTAGGCGCTACAACGCCGAAATGACCTTCTCACCCTTGTGTACTCACTATGTTGCTTTGGCGGGTCGACCTGGTTCCGACCCAGGCGGCCGGCGCCCCCAGCCTTAACTGGCCAGGACGCCCGGCTAAGTGCCCGCCAGTATACAAAACTCAAGAATTCATTTTGTGAAGTCCTGATATATCATTTAATTGATTAAAACTTTCAACAACGGATCTCTTGGTTCTGGCATCGATGAAGAACGCAGCGAAATGCGATAAGTAATGTGAATTGCAGAATTCAGTGAATCATCGAATCTTTGAACGCACATTGCGCCCTCTGGTATTCCGGAGGGCATGCCTGTTCGAGCGTCATTTCAACCCTCAAGCTCTGCTTGGTATTGGGCAACGTCCGCTGCCGGACGTGCCTTGAAGACCTCGGCGACGGCGTCCTAGCCTCGAGCGTAGTAGTAAAATATCTCGCTTTGGAGTGCTGGGCGACGGCCGCCGGACAATCGACCTTCGGTCTATTTTTCCAAGGTTGACCTCGGATCAGGTAGGGATACCCGCTGAACTTAAGCAT---

**4390 (MK448282)**

TTACTGAGTTACCGCTCTATAACCCTTTGTGAACATACCTCAACTGTTGCCTCGGCGGGCAGGCGGTAGCGATACCGCCTCCCCGGCGCCGTCCGCGGCGCGCCCGCCGGGGGATAACCAAACTCTGATTTAACGACGTTTCTTCTGAGTGGCACAAGCAAAACAATAATCAAAACTTTTAACAACGGATCTCTTGGTTCTGGCATCGATGAAGAACGCAGCGAAATGCGATAAGTAATGTGAATTGCAGAATTCAGTGAATCATCGAATCTTTGAACGCACATTGCGCCCGCCAGCATTCTGGCGGGCATGCCTGTTCGAGCGTCATTTCAACCCTCAAGCACTGCTTGGTGTTGGGGCCCTACGGCCGACGTAGGCCCCCAAAGACAGTGGCGGACCCTCCCGGAGCCTCCTTTGCGTAGTAACATTACCACCTCGCACTGGGATCCGGAGGGACTCTCCTGCCGTAAAAACCCCCCAAAAACATCACAAGGTTGACCTCGGATCAGGTAGGAATACCCGCTGAACTTAAGCAT---

**4392(MK448259)**

AACCTGCGGAGGGATCATTACCGAGTGAGGGCGCTCCGGCGCCCAACCTCCCAACCCTTTGATGAACCTCAACCTCATTCTGCCTCGGGGGCGACCCGGTCCTGCGAGGGTCCGCGGTCCCCCCGGCGGCGACCTTCAATAACCCTGCATCAATTGTCGTCAGATATTGAAGCGAATTCAATTAAAACTTTCAACAACGGATCTCTTGGTTCTGGCATCGATGAAGAACGCAGCGAAATGCGATAAGTAATGTGAATTGCAGAATTCAGTGAATCATCGAATCTTTGAACGCACATTGCGCCCCCTGGCATTCCGGGGGGCATGCCTGTTCGAGCGTCATTACACCAATCACGCCTGGCGTGGTATTGGGCGACGGGGCCGTCACACGCCCCGCGCCCCAATGACTCCCCGGCGGGACGGACCGTATCTCAGCGTTGTGCAAATGCCGCTGGCGAGGACGGGACGGTCGTGCCGTGAAAACCCTTCACCATCAGGTTGACCTCGGATCAGGTAGGGATACCCGCTGAACTTAAGCAT---

**4394(MK448260)**

AACCTGCGGAGGGATCATTAAAATAGGCCGATAAGAAGGCACTCTCCCCTCGGGGAGAGGGCGGGCCGTATCCCACACCCTTTGTGAACCACAACTCTGTTGCTTCGGGGGCGACCCTGCCGTCAGGCGGCGGTGCCCCCGGTGGCCTTCAACTAAACTCTGCATCTTTTGCGTCGGAGTCTTTTAAAGAATTAAACAAAACTTTCAACAACGGATCTCTTGGTTCTGGCATCGATGAAGAACGCAGCGAAATGCGATAAGTAATGTGAATTGCAGAATTCAGTGAATCATCGAATCTTTGAACGCACATTGCGCCCCGTGGTATTCCGCGGGGCATGCCTGTTCGAGCGTCATTTCACCACTCAAGCCTAGCTTGGTATTGGGCGCCGCGGCGATCTCCATCGCTGCGCGCCTTAAAGTCTCCGGCTGAGCAGTCTGTCTCCGAGCGTTGTGACACATGTCGCTAGGGAGTGCAGGTCTGCCGCGGCCGTTAAATCTTTATCAAAGGTTGACCTCGGATCAGGTAGGGATACCCGCTGAACTTAAGCAT---

**4396(MK432979)**

AACCTGCGGAGGGATCATTACCGAGTGTGGCGCTCCGGCGCCTCCCTCCAACCCCATGTCGAAACGACTCTGTTGCCTCGGGGGCGACCCGGCCTTCGGGCGTCGGGGCCCCCGGTGGACACCTTCATAACTCTTGCATCTCTTGCGTCTGAGTGATACATACAATCAATCAAAACTTTCAACAACGGATCTCTTGGTTCTGGCATCGATGAAGAACGCAGCGAAATGCGATAAGTAATGTGAATTGCAGAATTCAGTGAATCATCGAATCTTTGAACGCACATTGCGCCCCCTGGCATTCCGGGGGGCATGCCTGTTCGAGCGTCATTACACCACTCAAGCCTGGCTTGGTATTGAGCGCCGCGGCCTGCCCGCGCGCTCCAATGTCTCCGGCTGAGCCGTCCGTCTCTAAGCGTTGTGAATAGCGATCGC---

**4398 (MK448262)**

AACCTGCGGAGGGATCATTACCGAGTGGAGGGCCCCCGGGCCCGACCTCCAACCCTTCTGTCATCCGACCTCTGTTGCCTCGGGGGCGACCCGGCCCTCGCGCCGGGGCCCCCCGTGGACCGCCCTTGAAACTCTTGCATCTTTGCGTCTGAGTATGATTTTGAATCAAACAAAACTTTCAACAACGGATCTCTTGGTTCCAGCATCGATGAAGAACGCAGCGAAATGCGATAAGTAATGTGAATTGCAGAATTCAGTGAATCATCGAATCTTTGAACGCACATTGCGCCCCTCGGTATTCCGGGGGGCATGCCTGTTCGAGCGTCATTAACACCACTCAAGCCTCGTCTTGGTCTTGGGCGCCGCGGGGGCCACCCCCGCGCGCCTTGATGTCTCCGGCCGAGCCGTCCGTCTCTAAGCGTTGTGGAAACAATCCGCTTGCGAGCGCGGGGCGGTTCGTCAGCCGACCGAAACCTCTCTCTCAAGGTTGACCTCGGATCAGGTAGGGATACCCGCTGAACTTAAGCAT---

**4400(same colony morphology as 4334)**

AACCTGCGGAAGGATCATTACCTAGAGTTGCGGGCTTTGCCTGCCATCTCTTACCCATGTCTTTTGAGTACCTTCGTTTCCTCGGCGGGTTCGCCCGCCGGTTGGACAACACTTAAACCCTTTGTAATTGAAATCAGCGTCTGAAAAAACTTTAATAGTTACAACTTTCAACAACGGATCTCTTGGTTCTGGCATCGATGAAGAACGCAGCGAAATGCGATAAGTAGTGTGAATTGCAGAATTCAGTGAATCATCGAATCTTTGAACGCACATTGCGCCCCTTGGTATTCCATGGGGCATGCCTGTTCGAGCGTCATTTGTACCTTCAAGCTCTGCTTGGTGTTGGGTGTTTGTCTCGCCTCTGCGCGCAGACTCGCCTCAAAGCAATTGGCAGCCGGCGTATTGATTTCGGAGCGCAGTACATCTCGCGCTTTGCACTCATAACGACGACGTCCAAAAAGTACATTTTTTACACTCTTGACCTCGGATCAGGTAGGGATACCCGCTGAACTTAAGCAT---

**4402 (MK448268)**

AACCAGCGGAGGGATCATTACTGAGTTACCGCTCTATAACCCTTTGTGAACATACCTACAACTGTTGCTTCGGCGGGTAGGCCGTCCCGTGAAAAGGACGCCTCCCGGCCCGGCCCGGACCCCCCGCGGGAACGGACCCGGCGCCCGCCGGAGGATAACCAAACTCTGTTTTAACGACGTTTCTTCTGAGTGGCATAAGCAAAATAATCAAAACTTTTAACAACGGATCTCTTGGTTCTGGCATCGATGAAGAACGCAGCGAAATGCGATACGTAATGCGAATTGCAGAATTCAGTGAGTCATCGAATCTTTGAACGCACATTGCGCCCGCCAGCATTCTGGCGGGCATGCCTGTTCGAGCGTCATTTCAACCCTCAAGCTCTGCTTGGCGTTGGGGCTCTACGGTTGACGTAGGCCCCCAAAGGTAGTGGCGGACCCTCCCGGAGCCTCCTTTGCGTAGTAACATTTCGTCTCGCACTGGGATCCGGAGGGACTCTTGCCGTAAAACCCCCCAATTTTTCAAGGTTGACCTCGGATCAGGTAGGAATACCCGCTGAACTTAAGCAT---

**4404(same colony morphology as 4334)**

AACCTGCGGAAGGATCATTACCTAGAGTTGCGGGCTTTGCCTGCCATCTCTTACCCATGTCTTTTGAGTACCTTCGTTTCCTCGGCGGGTTCGCCCGCCGGTTGGACAACACTTAAACCCTTTGTAATTGAAATCAGCGTCTGAAAAAACTTTAATAGTTACAACTTTCAACAACGGATCTCTTGGTTCTGGCATCGATGAAGAACGCAGCGAAATGCGATAAGTAGTGTGAATTGCAGAATTCAGTGAATCATCGAATCTTTGAACGCACATTGCGCCCCTTGGTATTCCATGGGGCATGCCTGTTCGAGCGTCATTTGTACCTTCAAGCTCTGCTTGGTGTTGGGTGTTTGTCTCGCCTCTGCGCGCAGACTCGCCTCAAAGCAATTGGCAGCCGGCGTATTGATTTCGGAGCGCAGTACATCTCGCGCTTTGCACTCATAACGACGACGTCCAAAAAGTACATTTTTTACACTCTTGACCTCGGATCAGGTAGGGATACCCGCTGAACTTAAGCAT---

**4406(MK448251)**

AACCTGCGGAAGGATCATTACCTAGAGTTGTAGGCTTTGCCTGCTATCTCTTACCCATGTCTTTTGAGTACCTTACGTTTCCTCGGTGGGTTCGCCCACCGATTGGACAAATTTAAACCCTTTGCAGTTGAAATCAGCGTCTGAAAAAACTTAATAGTTACAACTTTCAACAACGGATCTCTTGGTTCTGGCATCGATGAAGAACGCAGCGAAATGCGATAAGTAGTGTGAATTGCAGAATTCAGTGAATCATCGAATCTTTGAACGCACATTGCGCCCCTTGGTATTCCATGGGGCATGCCTGTTCGAGCGTCATTTGTACCTTCAAGCTCTGCTTGGTGTTGGGTGTTTGTCTCCTGTAGACTCGCCTTAAAACAATTGGCAGCCGGCGTATTGATTTCGGAGCGCAGTACATCTCGCGCTTTGCACTCATAACGACGACATCCAAAAGTACATTTTTACACTCTTGACCTCGGATCAGGTAGGGATACCCGCTGAACTTAAGCAT---

**4408(MK448250)**

AACCTGCGGAAGGATCATTACATTCAGTAGCCTAGCTACTTGTTTACACCCTTGTTTTTTGCGTACCTATTGTTTCCTCGGCAGGCTTGCCTGCCGGCTGGACAAATTTATAACCTTTTTAAATCTTCAATCAGCGTCTGAATTATACATAATAATTACAACTTTCAACAACGGATCTCTTGGTTCTGGCATCGATGAAGAACGCAGCGAAATGCGATAAGTAGTGTGAATTGCAGAATTCAGTGAATCATCGAATCTTTGAACGCACATTGCGCCCCTTGGTATTCCATGGGGCATGCCTGTTCGAGCGTCATTTGTACCTTCAAGCTTTGCTTGGTGTTGGGCGTTTGTCTTTGTTATAAAGACTCGCCTTAAAGTTATTGGCAGCCAGTGTTTTTGGTAGTAAGCGCAGCACATTTTGCGTCTTGGTCCCTAAACAGCGGCATCCATGAAGCCATTTTCTCACTTTTGACCTCGGATCAGGTAGGGATACCCGCTGAACTTAAGCAT---

**4410(MK448276)**

AACCTGCGGAGGGATCATTACACAATAAAATACGAAGGCCGTTCGCGGCTGGACTATTTATTACCCTTGTCTTTTGCGCACTTGTTGTTTCCTGGGCGGGTTCGCTCGCCACCAGGACCACAATATAAACCTTTTTTATGCAGTTGCAATCAGCGTCAGTATAACAAATGTAAATCATTTACAACTTTCAACAACGGATCTCTTGGTTCTGGCATCGATGAAGAACGCAGCGAAATGCGATACGTAGTGTGAATTGCAGAATTCAGTGAATCATCGAATCTTTGAACGCACATTGCGCCCTTTGGTATTCCAAAGGGCATGCCTGTTCGAGCGTCATTTGTACCCTCAAGCTTTGCTTGGTGTTGGGCGTTTTTGTCCCCCCCAAAAGGGGGACTCGCCTTAAAAGGATTGGCAGCCGGCCTACTGGTTTCGCAGCGCAGCACATTTTTGCGCTTGCAATCAGCAAAAGAGGACGGCAATCCATCAAGACTCCTTCTCACGTTTGACCTCGGATCAGGTAGGGATACCCGCTGAACTTAAGCAT---

**4866(MK432970)**

AACCTGCGGAGGGATCATTACCGAGTTTACAACTCCCAAACCCCTGTGAACATACCAATTGTTGCCTCGGCGGATCAGCCCGCTCCCGGTAAAACGGGACGGCCCGCCAGAGGACCCCTAAACTCTGTTTCTATATGTAACTTCTGAGTAAAACCATAAATAAATCAAAACTTTCAACAACGGATCTCTTGGTTCTGGCATCGATGAAGAACGCAGCAAAATGCGATAAGTAATGTGAATTGCAGAATTCAGTGAATCATCGAATCTTTGAACGCACATTGCGCCCGCCAGTATTCTGGCGGGCATGCCTGTTCGAGCGTCATTTCAACCCTCAAGCCCCCGGGTTTGGTGTTGGGGATCGGCGAGCCCTTGCGGCAAGCCGGCCCCGAAATCTAGTGGCGGTCTCGCTGCAGCTTCCATTGCGTAGTAGTAAAACCCTCGCAACTGGTACGCGGCGCGGCCAAGCCGTTAAACCCCCAACTTCTGAA---

**4868(MK432980)**

AACCTGCGGAGGGATCATTAATGAGTTACTAAACTCCAAAACCCTTTGTGAACCTTACCGTCGTTTCCTCGGCGCGTGCTGCTGCTACCTGGAGCTACCCTGGAAGGCACCTACCCTGTAGTGGTTGTCTGCCAAGAGCTATCATGTCAGGCACCTACCTCTGTAGTGGTTGTCTGCCATGAGCTGTCTTGGCAGGCACCTACCCTGTAGTGGTTGTCTGCTAAGAGTTATCCTAGCAGGCACCTACCCTGTAGTGGTTGTCTACCCTGGAGCTACCCTGTAGCCGCGTGAAGGCCCGCCGAAGGACCGTTAAACTCTTGCTTCTACAACTGTATCTCTGAACACGTAACTGAAATGAGTTAAAACTTTCAACAACGGATCTCTTGGTTCTGGCATCGATGAAGAACGCAGCGAAATGCGATAAGTAATGTGAATTGCAGAATTCAGTGAATCATCGAATCTTTGAACGCACATTGCGCCCATTAGTATTCTAGTGGGCATGCCTATTCGAGCGTCATTTCAACCCTTAAGCCCTGTTGCTTAGTGTTGGGAGTCTACGGCTTCGGCGTAGTTCCTGAAAATCAGTGGCGGAGTTAGGGTACACTCTCAGCGTAGTAATATCTCTCGCTCGTGTGGTGGCCCTGGCTGCTGGCCGTAAAACCCCCTATTTTCTAGTGGTTGACCTCGGATTAGGTAGGAATACCCGCTGAACTTAAGCAT---

**4869 (same colony morphology as 4380)**

AACCAGCGGAGGGATCATTGCTGGAACGCGCTTCGGCGCACCCAGAAACCCTTTGTGAACTTATACCTATTGTTGCCTCGGCGTAGGCCGGCCTCTTCACTGAGGCCCCCTGGAAACAGGGAGCAGCCCGCCGGCGGCCAACCAAACTCTTGTTTCTACAGTGAATCTCTGAGTAAAAAACATAAATGAATCAAAACTTTCAACAACGGATCTCTTGGTTCTGGCATCGATGAAGAACGCAGCGAAATGCGATAAGTAATGTGAATTGCAGAATTCAGTGAATCATCGAATCTTTGAACGCACATTGCGCCCTCTGGTATTCCGGAGGGCATGCCTGTTCGAGCGTCATTTCAACCCTCAAGCCTGGCTTGGTGATGGGGCACTGCCTGTAAAAGGGCAGGCCCTGAAATCTAGTGGCGAGCTCGCCAGGACCCCGAGCGTAGTAGTTATATCTCGCTCTGGAAGGCCCTGGCGGTGCCCTGCCGTTAAACCCCCAACTTCTGAAAATTTGACCTCGGATCAGGTAGGAATACCCGCTGAACTTAAGCAT---

**4870(MK448277)**

AACCTGCGGAGGGATCATTACCGAGTTTACAACTCCCAAACCCCTGTGAACATACCACTTGTTGCCTCGGCGGATCAGCCCGCTCCCGGTAAAACGGGACGGCCCGCCAGAGGACCCCTAAACTCTGTTTCTATATGTAACTTCTGAGTAAAACCATAAATAAATCAAAACTTTCAACAACGGATCTCTTGGTTCTGGCATCGATGAAGAACGCAGCAAAATGCGATAAGTAATGTGAATTGCAGAATTCAGTGAATCATCGAATCTTTGAACGCACATTGCGCCCGCCAGTATTCTGGCGGGCATGCCTGTTCGAGCGTCATTTCAACCCTCAAGCACAGCTTGGTGTTGGGACTCGCGTTAATTCGCGTTCCCCAAATTGATTGGCGGTCACGTCGAGCTTCCATAGCGTAGTAGTAAAACCCTCGTTACTGGTAATCGTCGCGGCCACGCCGTTAAACCCCAACTTCTGAATGTTGACCTCGGATCAGGTAGGAATACCCGCTGAACTTAAGCAT---

**4871(MK432976)**

AACCTGCGGAAGGATCATTACTGAAATGTAATAACTTCTATTGAAAGGTTCCAGAGTAGGCGCTACAACGCCGAAATGACCTTCTCACCCTTGTGTACTCACTATGTTGCTTTGGCGGGTCGACCTGGTTCCGACCCAGGCGGCCGGCGCCCCCAGCCTTAACTGGCCAGGACGCCCGGCTAAGTGCCCGCCAGTATACAAAACTCAAGAATTCATTTTGTGAAGTCCTGATATATCATTTAATTGATTAAAACTTTCAACAACGGATCTCTTGGTTCTGGCATCGATGAAGAACGCAGCGAAATGCGATAAGTAATGTGAATTGCAGAATTCAGTGAATCATCGAATCTTTGAACGCACATTGCGCCCTCTGGTATTCCGGAGGGCATGCCTGTTCGAGCGTCATTTCAACCCTCAAGCTCTGCTTGGTATTGGGCAACGTCCGCTGCCGGACGTGCCTTGAAGACCTCGGCGACGGCGTCCTAGCCTCGAGCGTAGTAGTAAAATATCTCGCTTTGGAGTGCTGGGCGACGGCCGCCGGACAATCGACCTTCGGTCTATTTTTCCAAGGTTGACCTCGGATCAGGTAGGGATACCCGCTGAACTTAAGCAT---

**4872(MK432962)**

AACCTGCGGAAGGATCATTATCGTAGGGGCCTCGCCCCCTTCGAGATAGCACCCTTTGTTTATGAGCACCTCTCGTTTCCTCGGCAGGCTCGCCTGCCAACGGGGACCCACCACAAACCCATTGCAGTACAAGAAGTACACGTCTGAACAAAACAAAACAAACTATTTACAACTTTCAACAACGGATCTCTTGGTTCTGGCATCGATGAAGAACGCAGCGAAATGCGATAAGTAGTGTGAATTGCAGAATTCAGTGAATCATCGAATCTTTGAACGCACATTGCGCCCTTTGGTATTCCTTAGGGCATGCCTGTTCGAGCGTCATTTCAACCCTCAAGCCTAGCTTGGTGTTGGGCGTCTGTCCCGCCTCCGCGCGCCTGGACTCGCCTCAAAAGCATTGGCGGCCGGTTCCCAGCAGGCCACGAGCGCAGCAGAGCAAGCGCTGAAGTGGCTGCGGGTCGGCACACCATGAGCCCCCCCACACCAGAATTTTGACCTCGGATCAGGTAGGGATACCCGCTGAACTTAAGCAT---

**4873(MK448278)**

AACCAGCGGAGGGATCATTACCGAGTTTACAACTCCCAAACCCCTGTGAACATACCACTTGTTGCCTCGGCGGATCAGCCCGCTCCCGGTAAAACGGGACGGCCCGCCAGAGGACCCCTAAACTCTGTTTCTATATGTAACTTCTGAGTAAAACCATAAATAAATCAAAACTTTCAACAACGGATCTCTTGGTTCTGGCATCGATGAAGAACGCAGCAAAATGCGATAAGTAATGTGAATTGCAGAATTCAGTGAATCATCGAATCTTTGAACGCACATTGCGCCCGCCAGTATTCTGGCGGGCATGCCTGTTCGAGCGTCATTTCAACCCTCAAGCACAGCTTGGTGTTGGGACTCGCGTTAATTCGCGTTCCCCAAATTGATTGGCGGTCACGTCGAGCTTCCATAGCGTAGTAGTAAAACCCTCGTTACTGGTAATCGTCGCGGCCACGCCGTTAAACCCCAACTTCTGAATGTTGACCTCGGATCAGGTAGGAATACCCGCTGAACTTAAGCAT---

**4874(MK432977)**

AACCTGCGGAAGGATCATTACTGAAATGTAACAACTTCTATTGAAAGGTTCCAGAGTAGGCGCTACAACGCCGAAATGACCTTCTCACCCTTGTGTACTCACTATGTTGCTTTGGCGGGTCGACCTGGTTCCGACCCAGGCGGCCGGCGCCCCCAGCCTTAACTGGCCAGGACGCCCGGCTAAGTGCCCGCCAGTATACAAAACTCAAGAATTCATTTTGTGAAGTCCTGATATATCATTTAATTGATTAAAACTTTCAACAACGGATCTCTTGGTTCTGGCATCGATGAAGAACGCAGCGAAATGCGATAAGTAATGTGAATTGCAGAATTCAGTGAATCATCGAATCTTTGAACGCACATTGCGCCCTCTGGTATTCCGGAGGGCATGCCTGTTCGAGCGTCATTTCAACCCTCAAGCTCTGCTTGGTATTGGGCAACGTCCGCTGCCGGACGTGCCTTGAAGACCTCGGCGACGGCGTCCTAGCCTCGAGCGTAGTAGTAAAATATCTCGCTTTGGAGTGCTGGGCGACGGCCGCCGGACAATCGACCTTCGGTCTATTTTTCCAAGGTTGACCTCGGATCAGGTAGGGATACCCGCTGAACTTAAGCAT---

**4875 (MK448265)**

AACCTGCGGAAGGATCATTACCGAGTTGATTCGGGCTCCGGCCCGATCCTCCCACCCTTTGTGTACCTACCTCTGTTGCTTTGGCGGGCCGCGGTCCTCCGCGGCCGCCCCCCTCCCCGGGGGGGTGGCCAGCGCCCGCCAGAGGACCATCAAACTCCAGTCAGTAAACGATGCAGTCTGAAAAACATTTAATAAACTAAAACTTTCAACAACGGATCTCTTGGTTCTGGCATCGATGAAGAACGCAGCGAAATGCGATAAGTAATGTGAATTGCAGAATTCAGTGAATCATCGAATCTTTGAACGCACATTGCGCCCTTTGGTATTCCGAAGGGCATGCCTGTTCGAGCGTCATTACAACCCTCAAGCTCTGCTTGGTATTGGGCACCGTCCTTTGCGGGCGCGCCTCAAAGACCTCGGCGGTGGCGTCTTGCCTCAAGCGTAGTAGAACATACATCTCGCTTCGGAGCGCAGGGCGTCGCCCGCCGGACGAACCTTCTGAACTTTTCTCAAGGTTGACCTCGGATCAGGTAGGGATACCCGCTGAACTTAAGCAT---

**4876(MK432971)**

AACCAGCGGAGGGATCATTACCGAGTTTACAACTCCCAAACCCCTGTGAACATACCAATTGTTGCCTCGGCGGATCAGCCCGCTCCCGGTAAAACGGGACGGCCCGCCAGAGGACCCCTAAACTCTGTTTCTATATGTAACTTCTGAGTAAAACCATAAATAAATCAAAACTTTCAACAACGGATCTCTTGGTTCTGGCATCGATGAAGAACGCAGCAAAATGCGATAAGTAATGTGAATTGCAGAATTCAGTGAATCATCGAATCTTTGAACGCACATTGCGCCCGCCAGTATTCTGGCGGGCATGCCTGTTCGAGCGTCATTTCAACCCTCAAGCCCCCGGGTTTGGTGTTGGGGATCGGCGAGCCCTTGCGGCAAGCCGGCCCCGAAATCTAGTGGCGGTCTCGCTGCAGCTTCCATTGCGTAGTAGTAAAACCCTCGCAACTGGTACGCGGCGCGGCCAAGCCGTTAAACCCCCAACTTCTGAATGTTGACCTCGGATCAGGTAGGAATACCCGCTGAACTTAAGCAT---

**4877(MK432972)**

CTCCCAAACCCCTGTGAACATACCAATTGTTGCCTCGGCGGATCAGCCCGCTCCCGGTAAAACGGGACGGCCCGCCAGAGGACCCCTAAACTCTGTTTCTATATGTAACTTCTGAGTAAAACCATAAATAAATCAAAACTTTCAACAACGGATCTCTTGGTTCTGGCATCGATGAAGAACGCAGCAAAATGCGATAAGTAATGTGAATTGCAGAATTCAGTGAATCATCGAATCTTTGAACGCACATTGCGCCCGCCAGTATTCTGGCGGGCATGCCTGTTCGAGCGTCATTTCAACCCTCAAGCCCCCGGGTTTGGTGTTGGGGATCGGCGAGCCCTTGCGGCAAGCCGGCCCCGAAATCTAGTGGCGGTCTCGCTGCAGCTTCCATTGCGTAGTAGTAAAACCCTCGCAACTGGTACGCGGCGCGGCCAAGCCGTTAAACCCCCAACTTCTGAATGTTGACCTCGGATCAGGTAGGAATACCCGCTGAACTTAAGCAT---

**4878(MK432998)**

AACCTGCGGAAGGATCATTACCTAGAGTTGCGGGCTTTGCCTGCCATCTCTTACCCATGTCTTTTGAGTACCTTCGTTTCCTCGGCGGGTTCGCCCGCCGGTTGGACAACACTTAAACCCTTTGTAATTGAAATCAGCGTCTGAAAAAACTTTAATAGTTACAACTTTCAACAACGGATCTCTTGGTTCTGGCATCGATGAAGAACGCAGCGAAATGCGATAAGTAGTGTGAATTGCAGAATTCAGTGAATCATCGAATCTTTGAACGCACATTGCGCCCCTTGGTATTCCATGGGGCATGCCTGTTCGAGCGTCATTTGTACCTTCAAGCTCTGCTTGGTGTTGGGTGTTTGTCTCGCCTCTGCGCGCAGACTCGCCTCAAAGCAATTGGCAGCCGGCGTATTGATTTCGGAGCGCAGTACATCTCGCGCTTTGCACTCATAACGACGACGTCCAAAAAGTACATTTTTTACACTCTTGACCTCGGATCAGGTAGGGATACCCGCTGAACTTAAGCAT---

**4879(MK448283)**

AACCTGCGGAGGGATCATTACCGAGTTTACAACTCCCAAACCCCTGTGAACATACCACTTGTTGCCTCGGCGGATCAGCCCGCTCCCGGTAAAACGGGACGGCCCGCCAGAGGACCCCTAAACTCTGTTTCTATATGTAACTTCTGAGTAAAACCATAAATAAATCAAAACTTTCAACAACGGATCTCTTGGTTCTGGCATCGATGAAGAACGCAGCAAAATGCGATAAGTAATGTGAATTGCAGAATTCAGTGAATCATCGAATCTTTGAACGCACATTGCGCCCGCCAGTATTCTGGCGGGCATGCCTGTTCGAGCGTCATTTCAACCCTCAAGCACAGCTTGGTGTTGGGACTCGCGTTAATTCGCGTTCCCCAAATTGATTGGCGGTCACGTCGAGCTTCCATAGCGTAGTAGTAAAACCCTCGTTACTGGTAATCGTCGCGGCCACGCCGTTAAACCCCAACT---

**4883(MK432959)**

AACCTGCGGAGGGATCATTACAAGTGACCCCGGTCTAACCACCGGGATGTTCATAACCCTTTGTTGTCCGACTCTGTTGCCTCCGGGGCGACCCTGCCTTCGGGCGGGGGCTCCGGGTGGACACTTCAAACTCTTGCGTAACTTTGCAGTCTGAGTAAACTTAATTAATAAATTAAAACTTTTAACAACGGATCTCTTGGTTCTGGCATCGATGAAGAACGCAGCGAAATGCGATAAGTAATGTGAATTGCAGAATTCAGTGAATCATCGAATCTTTGAACGCACATTGCGCCCCCTGGTATTCCGGGGGGCATGCCTGTTCGAGCGTCATTTCACCACTCAAGCCTCGCTTGGTATTGGGCAACGCGGTCCGCCGCGTGCCTCAAATCGACCGGCTGGGTCTTCTGTCCCCTAAGCGTTGTGGAAACTATTCGCTAAAGGGTGCTCGGGAGGCTACGCCGTAAAACAAACCCATTTCTAAGGTTGACCTCGGATCAGGTAGGGATACCCGCTGAACTTAAGCAT---

**4884(MK432965)**

AACCAGCGGAGGGATCATTGCTGGAACGCGCTTCGGCGCACCCAGAAACCCTTTGTGAACTTATACCTATTGTTGCCTCGGCGTAGGCCGGCCTCTTCACTGAGGCCCCCTGGAAACAGGGAGCAGCCCGCCGGCGGCCAACCAAACTCTTGTTTCTACAGTGAATCTCTGAGTAAAAAACATAAATGAATCAAAACTTTCAACAACGGATCTCTTGGTTCTGGCATCGATGAAGAACGCAGCGAAATGCGATAAGTAATGTGAATTGCAGAATTCAGTGAATCATCGAATCTTTGAACGCACATTGCGCCCTCTGGTATTCCGGAGGGCATGCCTGTTCGAGCGTCATTTCAACCCTCAAGCCTGGCTTGGTGATGGGGCACTGCCTGTAAAAGGGCAGGCCCTGAAATCTAGTGGCGAGCTCGCCAGGACCCCGAGCGTAGTAGTTATATCTCGCTCTGGAAGGCCCTGGCGGTGCCCTGCCGTTAAACCCCCAACTTCTGAAAATTTGACCTCGGATCAGGTAGGAATACCCGCTGAACTTAAGCAT---

**4885(MK448284)**

CTCCCAAACCCCTGTGAACATACCACTTGTTGCCTCGGCGGATCAGCCCGCTCCCGGTAAAACGGGACGGCCCGCCAGAGGACCCCTAAACTCTGTTTCTATATGTAACTTCTGAGTAAAACCATAAATAAATCAAAACTTTCAACAACGGATCTCTTGGTTCTGGCATCGATGAAGAACGCAGCAAAATGCGATAAGTAATGTGAATTGCAGAATTCAGTGAATCATCGAATCTTTGAACGCACATTGCGCCCGCCAGTATTCTGGCGGGCATGCCTGTTCGAGCGTCATTTCAACCCTCAAGCACAGCTTGGTGTTGGGACTCGCGTTAATTCGCGTTCCCCAAATTGATTGGCGGTCACGTCGAGCTTCCATAGCGTAGTAGTAAAACCCTCGTTACTGGTAATCGTCGCGGCCACGCCGTTAAACCCCAACTTCTGAATGTTGACCTCGGATCAGGTAGGAATACCCGCTGAACTTAAGCAT---

**4886(MK448272)**

AACCTGCGGAGGGATCATTGCTGGAACGCGCTTCGGCGCACCCAGAAACCCTTTGTGAACTTATACCTATTGTTGCCTCGGCGTCAGGCCGGCCTCTTCACTGAGGCCCCCCGGAGACGGGGAGCAGCCCGCCGGCGGCCAACTAAACTCTTGTTTCTATAGTGAATCTCTGAGTAAAAAACATAAATGAATCAAAACTTTCAACAACGGATCTCTTGGTTCTGGCATCGATGAAGAACGCAGCGAAATGCGATAAGTAATGTGAATTGCAGAATTCAGTGAATCATCGAATCTTTGAACGCACATTGCGCCCCCTGGTATTCCGGGGGGCATGCCTGTTCGAGCGTCATTTCAACCCTCAAGCCTGGCTTGGTGATGGGGCACTGCTTCGAAAGGAGCAGGCCCTGAAATTCAGTGGCGAGCTCGCCAGGACCCCGAGCGTAGTAGTTATATCTCGCTTTGGAAGGCCCTGGCGGTGCCCTGCCGTTAAACCCCCAACTTCTGAAAATTTGACCTCGGATCAGGTAGGAATACCCGCTGAACTTAAGCAT---

**4889(MK432963)**

AACCTGCGGAAGGATCATTATCGTAGGGGCCTCGCCCCCTTCGAGATAGCACCCTTTGTTTATGAGCACCTCTCGTTTCCTCGGCAGGCTCGCCTGCCAACGGGGACCCCCCACAAACCCATTGCAGTACAAGAAGTACACGTCTGAACAAAACAAAACAAACCATTTACAACTTTCAACAACGGATCTCTTGGTTCTGGCATCGATGAAGAACGCAGCGAAATGCGATAAGTAGTGTGAATTGCAGAATTCAGTGAATCATCGAATCTTTGAACGCACATTGCGCCCTTTGGTATTCCTTAGGGCATGCCTGTTCGAGCGTCATTTCAACCCTCAAGCCTAGCTTGGTGTTGGGCGTCTGTCCCGCCTCCGCGCGCCTGGACTCGCCTCAAAAGCATTGGCGGCCGGTTCCCAGCAGGCCACGAGCGCAGCAGAGCAAGCGCTGAAGTGGCTGCGGGTCGGCGCACCATGAGCCCCCCCCACACCAGAA---

**4890(MK448256)**

AACCTGCGGAAGGATCATTACAATTCAGTAGCTTGCTACTGTTAGGAGGCGCTATAAGCCCGCATAGTTTTACTACTGATGAGCGGCAGGCCCTCTGACTGTACCCTTGTCTTTTGCGCACCCAAGTTTCCTTGGTGGGCCAGCCCGCCAACTGGACAACACTATAACCTTTTTTAATTATCAATCAGCGTCTGAAAATAACGTAATAATTACAACTTTCAACAACGGATCTCTTGGTTCTGGCATCGATGAAGAACGCAGCGAAATGCGATAAGTAGTGTGAATTGCAGAATTCAGTGAATCATCGAATCTTTGAACGCACATTGCGCCCCTTGGTATTCCATGGGGCATGCCTGTTCGAGCGTCATTTGTACCCTCAAGCTCTGCTTGGTGTTGGGTGTTTGTCCTCTCCTCTGCGGTTGGACTCGCCTTAAAGCAATTGGCAGCCAGTGTTTTTGGTATTGAAGCGCAGCACATTTTGCGCATCTAGCCGAAAATACTTGCGTCCATAAGCCTTATTTTCACTTTTGACCTCGGATCAGGTAGGGATACCCGCTGAACTTAAGCAT---

**4891(MK448261)**

AACCTGCGGAAGGATCATTACAATTCAGTAGCTTGCTACTGTTAGGAGGCGCTATAAGCCCGCATAGTTTTACTACTGATGAGCGGCAGGCCCTCTGACTGTACCCTTGTCTTTTGCGCACCCAAGTTTCCTTGGTGGGCCAGCCCGCCAACTGGACAACACTATAACCTTTTTTAATTATCAATCAGCGTCTGAAAATAACGTAATAATTACAACTTTCAACAACGGATCTCTTGGTTCTGGCATCGATGAAGAACGCAGCGAAATGCGATAAGTAGTGTGAATTGCAGAATTCAGTGAATCATCGAATCTTTGAACGCACATTGCGCCCCTTGGTATTCCATGGGGCATGCCTGTTCGAGCGTCATTTGTACCCTCAAGCTCTGCTTGGTGTTGGGTGTTTGTCCTCTCCTCTGCGGTTGGACTCGCCTTAAAGCAATTGGCAGCCAGTGTTTTTGGTATTGAAGCGCAGCACATTTTGCGCATCTAGCCGAAAATACTTGCGTCCATAAGCCTTA---

**4892(MK448255)**

AACCTGCGGAGGGATCATTACCAGAAGCCGCGTCGGCCGCAAGGCCGGCGCCTTCGCCCAACCCTTTGTGAACCGAACTCGATTGCCCCGGGGGACCCCGCGCCTGCGCGGGCCCCCGGCGGACACACCTCAACTCTTGTTGTCCCTGCCGTCTGAGTCGAAAACAAATGAAACAAAACTTTCAACAACGGATCTCTTGGTTCTGGCATCGATGAAGAACGCAGCGAAATGCGATAAGTAATGTGAATTGCAGAATTCAGTGAATCATCGAATCTTTGAACGCACATTGCGCCCCCTGGTATTCCGGGGGGCATGCCTGTTCGAGCGTCATTTCAACCAATCCAGCCCGGCTGGGTGTTGGGCGTCGCGGTCCCCCGCGCGCCTCAAAGTCCTCGGCGGAAGCCGCCCGTACCTCTGCGTGATGATACATCTTCGCTCGGGAGACGGGGGCGAGCGCCCGTACGCGTCGGCGGAGACGTCGATTTTCAGGTTGACCTCGGATCAGGTAGGGGTACCCGCTGAACTTAAGCAT---

**4893(MK432983)**

AACCTGCGGAGGGATCATTACTGAGTGAGGGCTCACGCCCGACCTCCAACCCTTTGTGAACCAAACTTGTTGCTTCGGGGGCGACCCTGCCGACGACTCCGTCGCCGGGCGCCCCCGGAGGTCTTCTAAACACTGCATCTTTGCGTCGGAGTTTAAACAAATTAAACAAAACTTTCAACAACGGATCTCTTGGTTCTGGCATCGATGAAGAACGCAGCGAAATGCGATAAGTAATGTGAATTGCAGAATTCAGTGAATCATCGAATCTTTGAACGCACATTGCGCCCTTTGGTATTCCGAAGGGCATGCCTGTTCGAGCGTCATTTCACCACTCAAGCCTGGCTTGGTATTGGGCGTCGCGGTTTTCCGCGCGCCTTAAAGTCTTCCGGCTGAGCTGTCCGTCTCTAAGCGTTGTGGATTTTTCAATTCGCTTCGGAGTGCGGATGGCCGCGGCCGTTAAATCTTTATTCAAAGGTTGACCTCGGATCAGGTAGGGATACCCGCTGAACTTAAGCAT---

**4895 (MK432993)**

AACCTGCGGAGGGATCATTACTGAGTTACCGCTCTATAACCCTTTGTGAACATACCTACAACTGTTGCTTCGGCGGGTAGGCCGTCCCCTGAAAAGGACGCCTCCCGGCCCGGACCGGACCCCCTGTGGGGCCGGACTCGGCGCCCGCCGGAGGATAACCAAACGCTATTTTAACGACGTTTCTTCTGAGTGGCATAAGCAAAATAATCAAAACTTTTAACAACGGATCTCTTGGTTCTGGCATCGATGAAGAACGCAGCGAAATGCGATAAGTAATGTGAATTGCAGAATTCAGTGAATCATCGAATCTTTGAACGCACATTGCGCCCGCCAGCATTCTGGCGGGCATGCCTGTTCGAGCGTCATTTCAACCCTCAAGCTCTGCTTGGTGTTGGGGCTCTACGGTCGACGTAGGCCCTCAAAGGTAGTGGCGGACCCTCCCGGAGCCTCCTTTGCGTAGTAACATTTCGTCTCGCACTGGGATCCGGAGGGACTCTTGCCGTAAAACCCCCCAATTTTTCAAGGTTGACCTCGGATCAGGTAGGAATACCCGCTGAACTTAAGCAT---

**4896(MK448285)**

CTCCCAAACCCCTGTGAACATACCACTTGTTGCCTCGGCGGATCAGCCCGCTCCCGGTAAAACGGGACGGCCCGCCAGAGGACCCCTAAACTCTGTTTCTATATGTAACTTCTGAGTAAAACCATAAATAAATCAAAACTTTCAACAACGGATCTCTTGGTTCTGGCATCGATGAAGAACGCAGCAAAATGCGATAAGTAATGTGAATTGCAGAATTCAGTGAATCATCGAATCTTTGAACGCACATTGCGCCCGCCAGTATTCTGGCGGGCATGCCTGTTCGAGCGTCATTTCAACCCTCAAGCACAGCTTGGTGTTGGGACTCGCGTTAATTCGCGTTCCCCAAATTGATTGGCGGTCACGTCGAGCTTCCATAGCGTAGTAGTAAAACCCTCGTTACTGGTAATCGTCGCGGCCACGCCGTTAAACCCCAACTTCTGAATGTTGACCTCGGATCAGGTAGGAATACCCGCTGAACTTAAGCAT---

**4898(MK448286)**

AACCTGCGGAGGGATCATTACCGAGTTTACAACTCCCAAACCCCTGTGAACATACCACTTGTTGCCTCGGCGGATCAGCCCGCTCCCGGTAAAACGGGACGGCCCGCCAGAGGACCCCTAAACTCTGTTTCTATATGTAACTTCTGAGTAAAACCATAAATAAATCAAAACTTTCAACAACGGATCTCTTGGTTCTGGCATCGATGAAGAACGCAGCAAAATGCGATAAGTAATGTGAATTGCAGAATTCAGTGAATCATCGAATCTTTGAACGCACATTGCGCCCGCCAGTATTCTGGCGGGCATGCCTGTTCGAGCGTCATTTCAACCCTCAAGCACAGCTTGGTGTTGGGACTCGCGTTAATTCGCGTTCCCCAAATTGATTGGCGGTCACGTCGAGCTTCCATAGCGTAGTAGTAAAACCCTCGTTACTGGTAATCGTCGCGGCCACGCCGTTAAACCCCAAC---

**4899 (MK432954)**

AACCTGCGGAGGGATCATTACACAAATATGAAGGCGGGCTGGAACCTCTCGGGGTTACAGCCTTGCTGAATTATTCACCCTTGTCTTTTGCGTACTTCTTGTTTCCTTGGTGGGTTCGCCCACCACTAGGACAAACATAAACCTTTTGTAATTGCAATCAGCGTCAGTAACAAATTAATAATTACAACTTTCAACAACGGATCTCTTGGTTCTGGCATCGATGAAGAACGCAGCGAAATGCGATAAGTAGTGTGAATTGCAGAATTCAGTGAATCATCGAATCTTTGAACGCACATTGCGCCCTTTGGTATTCCAAAGGGCATGCCTGTTCGAGCGTCATTTGTACCCTCAAGCTTTGCTTGGTGTTGGGCGTCTTGTCTCTAGCTTTGCTGGAGACTCGCCTTAAAGTAATTGGCAGCCGGCCTACTGGTTTCGGAGCGCAGCACAAGTCGCACTCTCTATCAGCAAAGGTCTAGCATCCATTAAGCCTTTTTTTCAACTTTTGACCTCGGATCAGGTAGGGATACCCGCTGAACTTAAGCAT---

**4900(MK432985)**

AACCTGCGGAGGGATCATTAAAGAGTTAAAAACAACTCCTAAACCCATGTGAAACCTACCTTTGTTGCCTCGGCAGGTCTGCAACTTACCCTGAGGGTACCTACCCTGTAGGTACCTTACCCGGTAGTTGCGGGCATAACCTGCCGGTGGTCTACTAAACTCTGTTTACTATGTCATTCTGAATAATATAACTAAATAAGTTAAAACTTTCAACAACGGATCTCTTGGTTCTGGCATCGATGAAGAACGCAGCGAAATGCGATAAGTAATGTGAATTGCAGAATTCAGTGAATCATCGAATCTTTGAACGCACATTGCGCCCATTAGTATTCTAGTGGGCATGCCTGTTCGAGCGTCATTTCAACCCTTAAGCCCTGTTGCTTAGCGTTGGGAGCCTACAGATACCCTCTGTAGTTCCTTAAAGTTAGTGGCGGAGTCGGTTTCACACTCTAGACGTAGTAAATTTTATCTCGCCTATAGATGAGCCGGTCCCTTGCCGTAAAACCCCCTAATTTCTAAAGGTTGACCTCGGATCAGGTAGGAATACCCGCTGAACTTAAGCAT---

**4901(MK448275)**

AACCTGCGGAAGGATCATTAATTTCCGACCAATATTTGTCGGTCTAGGGACATGGAAATTTTGAAAGCTTGGGTACACCCACTTTTAAAAGAGTACATTCCTTTCTACCCTTGTTCTTTTGTGCACTCATGTTTCCTCGGCAGGTTAGCCTGCCGATTGGACAATTTAAACCCTTTATTTCTCAATCAGCGTCTGAAAATAATCAATATTTACAACTTTCAACAACGGATCTCTTGGTTCTGGCATCGATGAAGAACGCAGCGAAATGCGATAAGTAGTGTGAATTGCAGAATTCAGTGAATCATCGAATCTTTGAACGCACATTGCGCCCCTTGGTATTCCATGGGGCATGCCTGTTCGAGCGTCATTTGTAAACTCAAGCTCTGCTTGGTGTTGGGTGGTTGTCTTTTTCAAAAGACTCGCCCTAAATACATTGGCAGCTAGTATATTAGTTTGAAGCGCAGCACATTTGCAATTCGAGCTGGTTATATTGGCATCCAGGAAGCATAACATCACGTTTGACCTCGGATCAG---

**4902 (MK432955)**

AACCTGCGGAGGGATCATTACACAAATATGAAGGCGGGCTGGAACCTCTCGGGGTTACAGCCTTGCTGAATTATTCACCCTTGTCTTTTGCGTACTTCTTGTTTCCTTGGTGGGTTCGCCCACCACTAGGACAAACATAAACCTTTTGTAATTGCAATCAGCGTCAGTAACAAATTAATAATTACAACTTTCAACAACGGATCTCTTGGTTCTGGCATCGATGAAGAACGCAGCGAAATGCGATAAGTAGTGTGAATTGCAGAATTCAGTGAATCATCGAATCTTTGAACGCACATTGCGCCCTTTGGTATTCCAAAGGGCATGCCTGTTCGAGCGTCATTTGTACCCTCAAGCTTTGCTTGGTGTTGGGCGTCTTGTCTCTAGCTTTGCTGGAGACTCGCCTTAAAGTAATTGGCAGCCGGCCTACTGGTTTCGGAGCGCAGCACAAGTCGCACTCTCTATCAGCAAAGGTCTAGCATCCATTAAGCC---

**4903(MK432996)**

AACCTGCGGAGGGATCATTACTGAGTTTACGCTCTATAACCCTTTGTGAACATACCTATAACTGTTGCTTCGGCGGGTAGGGTCTCCGTGACCCTCCCGGCCTCCCGCCCCCGGGCGGGTCGGCGCCCGCCGGAGGATAACCAAACTCTGATTTAACGACGTTTCTTCTGAGTGGTACAAGCAAATAATCAAAACTTTTAACAACGGATCTCTTGGTTCTGGCATCGATGAAGAACGCAGCGAAATGCGATAAGTAATGTGAATTGCAGAATTCAGTGAATCATCGAATCTTTGAACGCACATTGCGCCCGCCAGCATTCTGGCGGGCATGCCTGTTCGAGCGTCATTTCAACCCTCAAGCTCTGCTTGGTGTTGGGGCCCTACAGCTGATGTAGGCCCTCAAAGGTAGTGGCGGACCCTCCCGGAGCCTCCTTTGCGTAGTAACTTTACGTCTCGCACTGGGATCCGGAGGGACTCTTGCCGTAAAACCCCCAATTTTCCAAAGGTTGACCTCGGATCAGGTAGGAATACCCGCTGAACTTAAGCAT---

**4904 (MK432956)**

AACCTGCGGAGGGATCATTACACAAATATGAAGGCGGGCTGGAACCTCTCGGGGTTACAGCCTTGCTGAATTATTCACCCTTGTCTTTTGCGTACTTCTTGTTTCCTTGGTGGGTTCGCCCACCACTAGGACAAACATAAACCTTTTGTAATTGCAATCAGCGTCAGTAACAAATTAATAATTACAACTTTCAACAACGGATCTCTTGGTTCTGGCATCGATGAAGAACGCAGCGAAATGCGATAAGTAGTGTGAATTGCAGAATTCAGTGAATCATCGAATCTTTGAACGCACATTGCGCCCTTTGGTATTCCAAAGGGCATGCCTGTTCGAGCGTCATTTGTACCCTCAAGCTTTGCTTGGTGTTGGGCGTCTTGTCTCTAGCTTTGCTGGAGACTCGCCTTAAAGTAATTGGCAGCCGGCCTACTGGTTTCGGAGCGCAGCACAAGTCGCACTCTCTATCAGCAAAGGTCTAGCATCCATTAAG---

**4905(MK448270)**

AACCTGCGGAGGGATCATTACTGAGTTATCTAAACTCCAACCCTATGTGAACTTACCGCCGTTGCCTCGGCGGGCCGCGTTCGCCCTGTAGTTTACTACCTGGCGGCGCGCTACAGGCCCGCCGGTGGACTGCTAAACTCTGTTATATATACGTATCTCTGAATGCTTCAACTTAATAAGTTAAAACTTTCAACAACGGATCTCTTGGTTCTGGCATCGATGAAGAACGCAGCGAAATGCGATAAGTAATGTGAATTGCAGAATTCAGTGAATCATCGAATCTTTGAACGCACATTGCGCCCATTAGTATTCTAGTGGGCATGCCTGTTCGAGCGTCATTTCAACCCTTAAGCCCCTGTTGCTTAGCGTTGGGAATCTAGGTCTCCAGGGCCTAGTTCCCCAAAGTCATCGGCGGAGTCGGAGCGTACTCTCAGCGTAGTAATACCATTCTCGCTTTTGCAGTAGCCCCGGCGGCTTGCCGTAAAACCCCTATATCTTTAGTGGTTGACCTCGAATCAGGTAGGAATACCCGCTGAACTTAAGCAT---

**4906(MK432982)**

AACCTGCGGAAGGATCATTAACGAGTTAATTGAACGGGTTGTAGCTGGTCCTCGCAAGAGGGCATGTGCACACCTGGCTCATCCACTCTTCAACCTCTGTGCACTTATTGTAGGTCGGCAGAAGAGTAAGCTTTCGGGCTTGCTTGGAAGCCTTCCTATGTTTCATTACAAACGCTTCAGTTTAAGAATGTAATCTCTGCGTATAACGCAATTATATACAACTTTCAGCAACGGATCTCTTGGCTCTCGCATCGATGAAGAACGCAGCGAAATGCGATAAGTAATGTGAATTGCAGAATTCAGTGAATCATCGAATCTTTGAACGCACCTTGCGCTCCCTGGTATTCCGGGGAGCATGCCTGTTTGAGTGTCATGGTATTCTCAACCTTCATAACTTTTTGTTATCGAAGGCTTGGATTTGGAGGCTTGTGCTGGCTTTTGTGAGTCGGCTCCTCTGAAATGCATTAGCGTGAGTGTAACGGATCGCTTCGGTGTGATAATTATCTGCGCCGTGGTCGTGAAGTGACATAAGCTTGCGCTTCTAGCCGTCCTTCAGTTGGACAATATCTTTGACATCTGACCTCAAATCAGGTAGGACTACCCGCTGAACTTAAGCAT---

**4907 (MK448271)**

AACCAGCGGAGGGATCATTACTGAGTTATCTAAACTCCAACCCTATGTGAACTTACCGCCGTTGCCTCGGCGGGCCGCGTTCGCCCTGTAGTTTACTACCTGGCGGCGCGCTACAGGCCCGCCGGTGGACTGCTAAACTCTGTTATATATACGTATCTCTGAATGCTTCAACTTAATAAGTTAAAACTTTCAACAACGGATCTCTTGGTTCTGGCATCGATGAAGAACGCAGCGAAATGCGATAAGTAATGTGAATTGCAGAATTCAGTGAATCATCGAATCTTTGAACGCACATTGCGCCCATTAGTATTCTAGTGGGCATGCCTGTTCGAGCGTCATTTCAACCCTTAAGCCCCTGTTGCTTAGCGTTGGGAATCTAGGTCTCCAGGGCCTAGTTCCCCAAAGTCATCGGCGGAGTCGGAGCGTACTCTCAGCGTAGTAATACCATTCTCGCTTTTGCAGTAGCCCCGGCGGCTTGCCGTAAAACCCCTATATCTTTAGTGGTTGACCTCGAATCAGGTAGGAATACCCGCTGAACTTAAGCAT---

**4908 (MK432988)**

AACCTGCGGAGGGATCATTACTGAGTTACCGCTCTATAACCCTTTGTGAACATACCTACAACTGTTGCTTCGGCGGGTAGGCCGTCCCCTGAAAAGGACGCCTCCCGGCCCGGACCGGACCCCCTGTGGGGCCGGACTCGGCGCCCGCCGGAGGATAACCAAACGCTATTTTAACGACGTTTCTTCTGAGTGGCATAAGCAAAATAATCAAAACTTTTAACAACGGATCTCTTGGTTCTGGCATCGATGAAGAACGCAGCGAAATGCGATAAGTAATGTGAATTGCAGAATTCAGTGAATCATCGAATCTTTGAACGCACATTGCGCCCGCCAGCATTCTGGCGGGCATGCCTGTTCGAGCGTCATTTCAACCCTCAAGCTCTGCTTGGTGTTGGGGCTCTACGGTCGACGTAGGCCCTCAAAGGTAGTGGCGGACCCTCCCGGAGCCTCCTTTGCGTAGTAACATTTCGTCTCGCACTGGGATCCGGAGGGACTCTTGCCGTAAAACCCCCCAA---

**4909 (MK432957)**

AACCAGCGGAGGGATCATTATTAGAAGCCGAAAGGCTACTTAAAACCATCGCGAACTCGTCCAAGTTGCTTCGGCGGCGCGGCCTCCCTCACGGGGGCGCCGCAGCCCCGCCTCTCCGGAGGTGTGGGGCGCCCGCCGGAGGTACGAAACTCTGTATTATAGTGGCATCTCTGAGTAAAAAACAAATAAGTTAAAACTTTCAACAACGGATCTCTTGGTTCTGGCATCGATGAAGAACGCAGCGAAATGCGATAAGTAATGTGAATTGCAGAATTCAGTGAATCATCGAATCTTTGAACGCACATTGCGCCCGCTAGTACTCTAGCGGGCATGCCTGTTCGAGCGTCATTTCAACCCTCAAGCCCTGCTTGGTGTTGGGGCCCTACGGCTGCCGTAGGCCCTGAAAGGAAGTGGCGGGCTCGCTACAACTCCGAGCGTAGTAATTCATTATCTCGCTAGGGACGTTGCGGCGCGCTCCTGCCGTTAAAGACCATCTTTAACTCAAGGTTGACCTCGGATCAGGTAGGAATACCCGCTGAACTTAAGCAT---

**4914(MK432981)**

AACCTGCGGAGGGATCATTATAGAGTTTTCTAAACTCCCAACCCATGTGAACTTACCATTGTTGCCTCGGCAGAAGCTGCTCGGCGCGCCTTACCTTGGAACGGCCTACCCTGTAGCGCCTTACCCTGGAACGGCTTACCCTGCAACGGCTGCCGGTGGACTACCAAACTCTTGTTATTTTATGGTTATCTGAGCGTCTTATTTTAATAAGTCAAAACTTTCAACAACGGATCTCTTGGTTCTGGCATCGATGAAGAACGCAGCGAAATGCGATAAGTAATGTGAATTGCAGAATTCAGTGAATCATCGAATCTTTGAACGCACATTGCGCCCATTAGTATTCTAGTGGGCATGCCTGTTCGAGCGTCATTTCAACCCTTAAGCCTAGCTTAGTGTTGGGAGCCTACTGCTTTTGCTAGCTGTAGCTCCTGAAATACAACGGCGGATCTGCGATATCCTCTGAGCGTAGTAATTTTTATCTCGCTTTTGACTGGAGTTGCAGCGTCTTTAGCCGCTAAACCCCCCAATTTTTAATGGTTGACCTCGGATCAGGTAGGAATACCCGCTGAACTTAAGCAT---

**4915(MK448274)**

AACCAGCGGAGGGATCATTGCTGGAACGCGCTTCGGCGCACCCAGAAACCCTTTGTGAACTTATACCTATTGTTGCCTCGGCGCAGGCCGGCCTCTTCACTGAGGCCCCCTGGAGACAGGGAGCAGCCCGCCGGCGGCCAACTAAACTCTTGTTTCTTTAGTGAATCTCTGAGTAAAAACATAAATGAATCAAAACTTTCAACAACGGATCTCTTGGTTCTGGCATCGATGAAGAACGCAGCGAAATGCGATAAGTAATGTGAATTGCAGAATTCAGTGAATCATCGAATCTTTGAACGCACATTGCGCCCTCTGGTATTCCGGAGGGCATGCCTGTTCGAGCGTCATTTCAACCCTCAAGCCTGGCTTGGTGATGGGGCACTGCTCTCTAGCGGGAGCAGGCCCTGAAATCTAGTGGCGAGCTCGCCAGGACCCCGAGCGTAGTAGTTATATCTCGTTCTGGAAGGCCCTGGCGGTGCCCTGCCGTTAAACCCCCAACTTCTGAAAATTTGACCTCGGATCAGGTAGGAATACCCGCTGAACTTAAGCAT---

**4916(MK432984)**

AACCTGCGGAAGGATCATTAACGAGTTGAACGGGGTTGTAGCTGGCCTTCACGGGCATGTGCACGCCTCACTCATCCACTCTACACCTGTGCACTTACTGTGGGTTTCGAGAGGCCGCGCTTGCGTGGTTGATCGGGCTCACGTCTATTACAAACTCTTCAGTATCAGAATGTGTATCGCGATGTAACGCATCTATATACAACTTTCAGCAACGGATCTCTTGGCTCTCGCATCGATGAAGAACGCAGCGAAATGCGATAAGTAATGTGAATTGCAGAATTCAGTGAATCATCGAATCTTTGAACGCACCTTGCGCTCCTTGGTATTCCGAGGAGCATGCCTGTTTGAGTGTCGTGTAATTCTCAACCTATAAATCCTTGTGGTTTTTAGGCTTGGACTTGGAGGCTTTTGCTGGCTTTACCGTCGGCTCCTCTTAAACGCATTAGCTTGATTCCTTGCGGATCGGCTCTCAGTGTGATAATTATCTGCGCTGTGACCGTGAAGCGTTTGGCGAGCTTCTAACCGTCTCTTTGAGACAAACACTTTGACATCTGACCTCAAATCAGGTAGGACTACCCGCTGAACTTAAGCAT---

**4917(MK440618)**

AACCTGCGGAAGGATCATTAATGAGTTTTTTAAAGTAAGCTTGATGCTGGTGGGTCTCTGGACTTGCATGTGCTCAGTTTGCGCTCATCCATCTCACACCTGTGCACTTACTGAAGAGAGAGAGGGAGAGGGAGAGTGGTTTATTCGTTTATTCATTTATTCGTGTATTCAACTCAAAGTCTTCAATCTCTCTTTTGACTTTATAATAAACAACTATATTGTTTGTGTAGAATGCATTAGCCTCATTGTAGGTGAAATAACTATACAACTTTCAACAACGGATCTCTTGGCTCTCGCATCGATGAAGAACGCAGCGAAATGCGATAAGTAATGTGAATTGCAGAATTCAGTGAATCATCGAATCTTTGAACGCACCTTGCACTCCTTGGTATTCCGAGGAGTATGCCTGTTTGAGTGTCATGTTAATCTCAATACAACATTTTTTGTAACTAAAAAGTGTTGATATTGGACTTGGGGACTGCTGGCGTAAGTCGGCTTCTCTTGAATGCATTAGCTGGGCTTTTGCTCGAGTAATTGGTGTAATAGTTTCTAACATTCACCGTTTACACTTGCTAATAGAGTCTGCTTCTAATCGTCTTGTAATGAGACAAAGACTTAACTTTGACCTTTGGCCTCAAATCAGGTAGGACTACCCGCTGAACTTAAGCAT---

**4918(MK432997)**

AACCTGCGGAGGGATCATTGCTGGAACGCGCCCCAGGCGCACCCAGAAACCCTATGTGAACTCATACCTCTGTTGCCTCGGCGCTGCTGGCCTTTCTGGGCCCTTTACTGCTGTAGTGGTAGAGAGAAGGCACGCCGGTGGCCAAAACAACCCTGTTTTTATACTGAAACTCTGAGCAAAAAACAAAATGAATCAAAACTTTCAACAACGGATCTCTTGGTTCTGGCATCGATGAAGAACGCAGCGAAATGCGATAAGTAATGTGAATTGCAGAATTCAGTGAATCATCGAATCTTTGAACGCACATTGCGCCCTCTGGCATTCCGGAGGGCATGCCTGTTCGAGCGTCATTTCACCCCTCAAGCACTGCTTGGTGTTGGGGCACTGCTACACCCAGGAGCAGGCCCTCAAATTCAGCGGCGAGCTCGCCAGGACCCCGAGCGCAGTAGTTAAACCCTCGCTCTGGAAGGCCCTGGCGGTGCCCTGCCGTTAAACCCCCAACTTCTGAAAATTTGACCTCGGATCAGGTAGGAATACCCGCTGAACTTAAGCAT---

**4920 (MK432966)**

AACCTGCGGAGGGATCATTGCTGGAACGCGCTTCGGCGCACCCAGAAACCCTTTGTGAACTTATACCTATTGTTGCCTCGGCGTAGGCCGGCCTCTTCACTGAGGCCCCCTGGAAACAGGGAGCAGCCCGCCGGCGGCCAACCAAACTCTTGTTTCTACAGTGAATCTCTGAGTAAAAAACATAAATGAATCAAAACTTTCAACAACGGATCTCTTGGTTCTGGCATCGATGAAGAACGCAGCGAAATGCGATAAGTAATGTGAATTGCAGAATTCAGTGAATCATCGAATCTTTGAACGCACATTGCGCCCTCTGGTATTCCGGAGGGCATGCCTGTTCGAGCGTCATTTCAACCCTCAAGCCTGGCTTGGTGATGGGGCACTGCCTGTAAAAGGGCAGGCCCTGAAATCTAGTGGCGAGCTCGCCAGGACCCCGAGCGTAGTAGTTATATCTCGCTCTGGAAGGCCCTGGCGGTGCCCTGCCGTTAAACCCCCAACTTCTGAAAATTTGACCTCGGATCAGGTAGGAATACCCGCTGAACTTAAGCAT---
